# STING agonist profiling by nucleotide library defines structural determinants of cyclic dinucleotide recognition

**DOI:** 10.1016/j.isci.2026.117179

**Published:** 2026-08-13

**Authors:** Indra Bekere, Yuliia Hubarzhevska, Sabrina V. Egender, Patrick K. Quoika, Rupert Öllinger, Marie Rose Schrimpf, Roland Rad, Martin Zacharias, Carina C. de Oliveira Mann

**Affiliations:** 1 Department of Bioscience, TUM School of Natural Sciences, Technical University of Munich, 85748 Garching, Germany; 2 Institute of Molecular Oncology and Functional Genomics and Department of Medicine II, School of Medicine, Technical University of Munich, 81675 Munich, Germany; 3 Gene Center and Department of Biochemistry, Ludwig-Maximilians-Universität München, 81377 Munich, Germany; 4 Cluster for Nucleic Acid Sciences and Technologies (NUCLEATE), 81377 Munich, Germany

**Keywords:** cGAS-STING, cyclic nucleotides, innate immunity, second messengers

## Abstract

Stimulator of interferon genes (STING) is the principal mammalian receptor for cyclic dinucleotides (CDNs) and a central hub of innate immune signaling. CDN analogues and small-molecule STING agonists are in active development for cancer immunotherapy and as vaccine adjuvants, although clinical translation has proven challenging. Here, we systematically profile STING nucleotide preferences by treating THP-1 monocytes and pancreatic cancer cells with a structurally diverse nucleotide library. We identify 27 CDNs that activate STING, while non-CDN nucleotides failed to elicit detectable responses, underscoring the selectivity of STING for its ligands. We demonstrate that STING’s preference for 2ʹ3ʹ-linked CDNs underlies its capacity to accommodate purine-pyrimidine hybrid nucleobases. Molecular dynamics simulations reveal that nucleobase position relative to the 2ʹ3ʹ linkage is a critical determinant of STING engagement. In addition, we show STING activation by diverse non-hydrolysable c-di-AMP and cGAMP isomers, thereby expanding the opportunities for designing STING agonists for therapeutic applications.

## Highlights

• Systematic screen of STING nucleotide preferences with a diverse nucleotide library
• STING is activated by 27 cyclic dinucleotides in monocytes and pancreatic cancer cells
• STING prefers cyclic dinucleotides with purine bases and 2′3′ phosphodiester linkage
• STING tolerates pyrimidine-containing CDNs only with a 2′3′ linkage

## Graphical abstract

**Figure:**
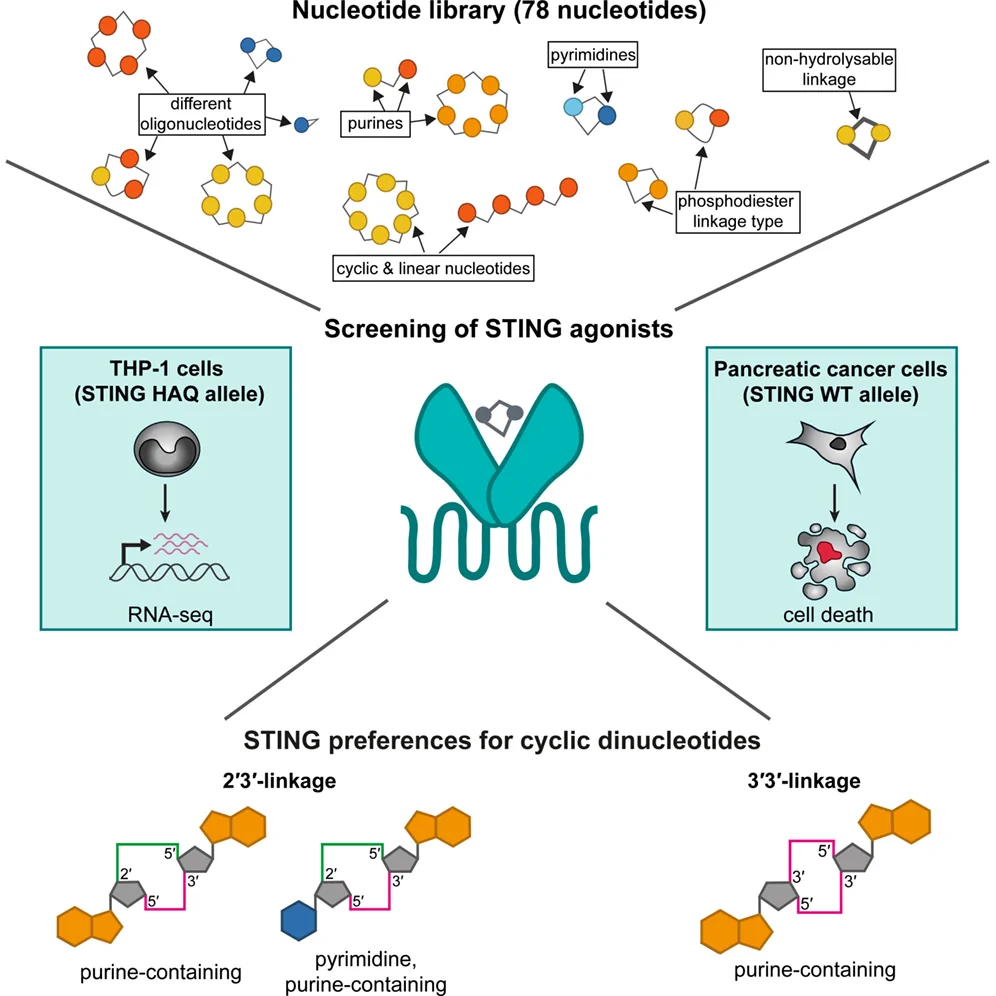

## Teaser

Biochemistry; Genetics; Molecular structure

## Introduction

In metazoans, recognition of cytosolic dsDNA by cyclic GMP-AMP synthase (cGAS) triggers synthesis of the second messenger 2′3′-cyclic GMP-AMP (2′3ʹ-cGAMP), a CDN carrying an unusual mixed 2′5′–3′5′ phosphodiester linkage and the most potent endogenous STING ligand.^1^^,^^2^^,^^3^^,^^4^ Upon ligand binding, STING undergoes major conformational changes and traffics from the endoplasmic reticulum to the Golgi, eliciting several downstream signaling outcomes: the canonical IRF3-driven type I interferon response,^5^^,^^6^^,^^7^^,^^8^ NF-κB-driven production of pro-inflammatory cytokines,^9^^,^^10^^,^^11^^,^^12^ LC3-associated autophagy and lysosomal remodeling, as well as promotion of lysosomal biogenesis.^13^^,^^14^^,^^15^^,^^16^^,^^17^

The potency of STING-dependent immune activation has made CDNs attractive candidates for pharmacological development, particularly as cancer immunotherapy and vaccine adjuvants.^18^^,^^19^^,^^20^^,^^21^ From a therapeutic standpoint, understanding STING’s cyclic nucleotide preferences is essential for agonist design, yet the clinical path has proven unexpectedly difficult. Natural CDN agonists face significant pharmacological obstacles: 2′3′-cGAMP is negatively charged rendering it non-cell permeable and dependent on specific transporters for uptake, which vary across cell types.^22^^,^^23^^,^^24^ 2′3′-cGAMP is also subjected to cleavage by extracellular enzymes.^25^^,^^26^^,^^27^ Ectonucleotide pyrophosphate phosphodiesterase 1 (ENPP1) is even exploited by cancer cells to degrade the immunotransmitter 2′3′-cGAMP and to generate immunosuppressive adenosine, thereby dampening anticancer immune responses within the tumor microenvironment.^28^ These liabilities motivated development of stabilized, non-hydrolysable CDN analogues, such as ADU-S100 (the Rp, Rp diastereomer of 2′3′-*c*-di-AMPSS) and synthetic STING agonists including diABZI and E7766.^26^^,^^29^^,^^30^^,^^31^ Among CDN-class agonists, ADU-S100 and MK-1454 demonstrated limited clinical efficacy in Phase 1/2 trials, and no STING agonist has yet advanced to Phase 3.^32^^,^^33^^,^^34^^,^^35^ These outcomes have nonetheless informed a clearer mechanistic understanding of the requirements for effective STING-targeted therapy, and multiple programs continue to pursue improved strategies. Several interconnected biological challenges underlie this translational gap between preclinical promise and clinical efficacy (reviewed elsewhere in the study by Temizoz and Ishii^18^), including STING’s nucleotide selectivity, which is modulated by naturally occurring human polymorphisms. Multiple single nucleotide polymorphisms (SNPs) in human STING create functionally distinct population variants: both the wild-type (WT) and HAQ (H72/A230/Q293) variant respond to CDNs with 2′3′ and 3′3′ linkages, while the R232H variant is unresponsive to 3′3′-linked CDNs.^3^^,^^29^^,^^36^ Rational optimization of next-generation STING agonists and delivery platforms therefore requires a deeper mechanistic and STING allele-specific understanding of which structural determinants, such as nucleobase identity, phosphodiester linkage, and chemical modifications determine agonist activity.

The chemical space of CDN second messengers has expanded considerably in recent years. Bacterial cGAS-like enzymes synthesize a chemically diverse repertoire of CDNs that differ in phosphodiester linkage geometry and nucleobase composition, including mixed purine-pyrimidine molecules, and activate STING homologs in bacteria, Drosophila, and cnidarians as part of antiviral defense.^37^^,^^38^^,^^39^^,^^40^^,^^41^ Notably, STING was first characterized as a receptor for bacterial CDNs (c-di-GMP, c-di-AMP, and 3′3′-cGAMP) before its endogenous metazoan ligand 2′3′-cGAMP was identified.^1^^,^^2^^,^^3^^,^^42^^,^^43^^,^^44^^,^^45^ This evolutionary precedent raises the question of whether additional, yet untested, CDNs can engage human STING and shape its downstream signaling.

Bacterial CDNs also operate in a wide range of pathways beyond antiviral defense, mediating diverse signaling functions outside the cGAS-STING axis, including roles in biofilm formation and metabolic regulation in bacteria, as well as differentiation in amoeba.^46^^,^^47^ Additional evidence for alternative CDN-mediated signaling pathways comes from studies in mice, where c-di-AMP not only activates STING but also signals through the cytosolic oxidoreductase RECON.^48^ This raises the question of whether humans harbor additional receptors or pathways dedicated to detecting cyclic oligo-nucleotides beyond STING-dependent 2′3′-cGAMP signaling.

STING exhibits a binding and activation pattern that is highly selective for nucleotides with a specific nucleobase composition and phosphodiester linkage types.^4^^,^^49^ Despite extensive structural and biochemical work, no comprehensive cellular analysis has evaluated the full spectrum of CDN classes, including recently discovered bacterial and invertebrate CDNs containing pyrimidine bases and non-canonical linkages,^37^^,^^38^^,^^39^^,^^40^^,^^41^ for their ability to activate endogenous STING in a single, consistent cellular system. Here, we report systematic functional profiling of STING (HAQ allele)-dependent transcriptional responses in human THP-1 cells and STING (WT allele)-dependent cell death in pancreatic cancer cell line (DANG) by a library containing 78 nucleotide compounds. We identify a subset of CDNs that robustly activate STING and define the common structural features underlying STING’s nucleobase and linkage preferences. This approach enabled unbiased structure-activity profiling across a broad chemical space of CDNs, establishing the structural requirements for STING activation. Notably, we also show that many bacterial CDNs fail to elicit any signaling in the selected cell lines, further underscoring the high specificity of CDN recognition and the strong selectivity of human STING for its endogenous ligand, 2′3′-cGAMP. In addition, we demonstrate how CDN modifications and their isomers, such as phosphorothioate-containing CDNs, that confer resistance to hydrolysis, modulate STING activation, providing insights with direct relevance for the development of therapeutic STING agonists. Mechanistically, we dissect the contribution of nucleobase identity at each position, an especially underexplored determinant of CDN recognition. We demonstrate that 2′3′-cUAMP and 3′2′-cUAMP exhibit dramatically different STING activation profiles despite differing only in nucleobase positioning. Molecular dynamics (MD) simulations indicate reduced 3′2′-cUAMP interaction with STING caused by subtle conformational changes, however, no clearly defined altered interaction pattern can be observed.

## Results

### Nucleotide library screen reveals selective signaling by purine-containing cyclic dinucleotides

**Figure 1:**
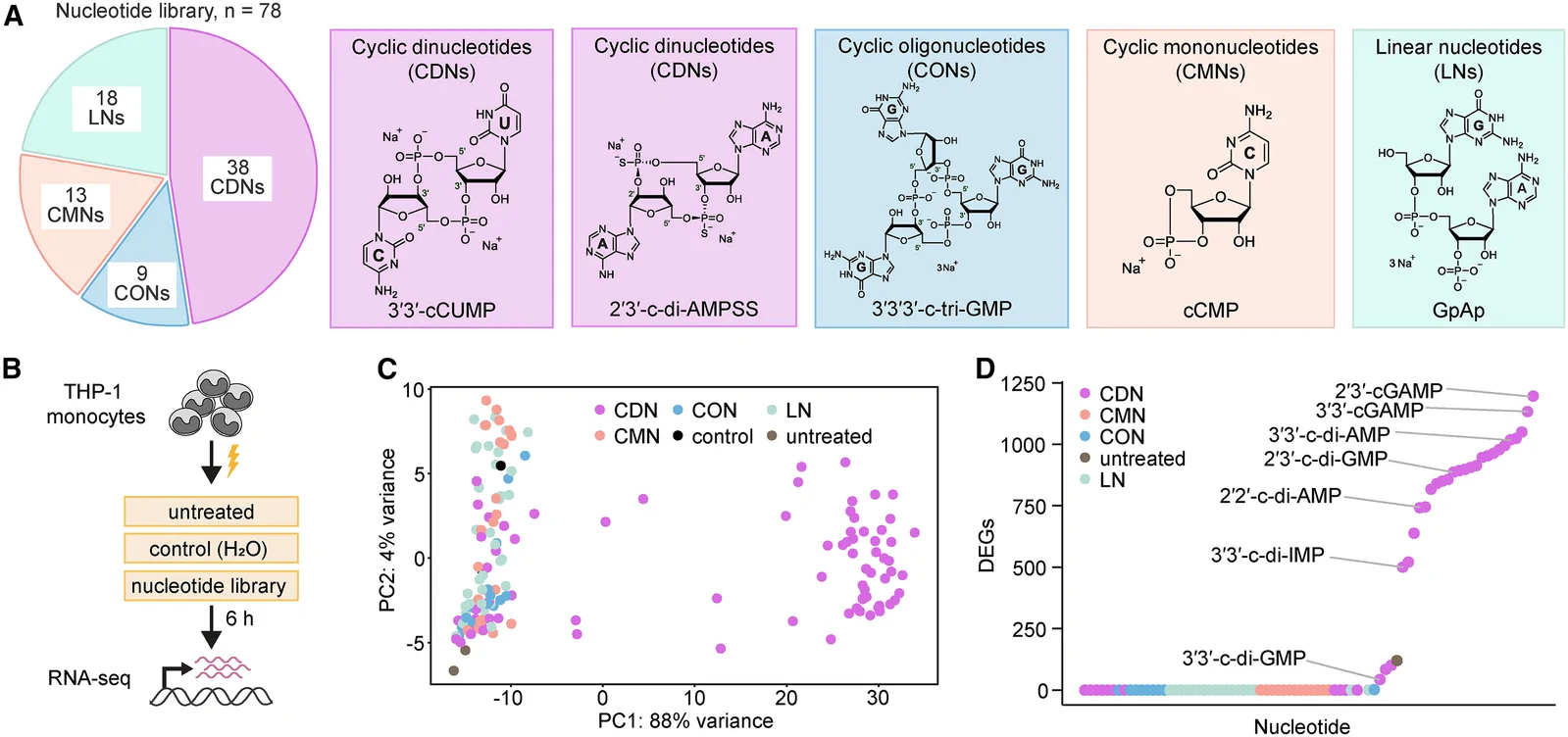
Nucleotide library screen reveals selective signaling by purine-containing cyclic dinucleotides (A) Composition of the nucleotide library with representative nucleotides shown. (B) Experimental setup. THP-1 monocytes were electroporated with 600 nM of each nucleotide in the library in duplicate or water as a control. Cells were harvested for RNA-seq analysis 6 h after electroporation. Untreated cells without electroporation were used to monitor gene expression changes due to electroporation alone. (C) Principal component analysis of transcript counts from RNA-seq analysis showing all the analyzed samples and their respective nucleotide type. (D) Ranking of nucleotides by the number of differentially expressed genes (log2 fold change ≥ [1] and adjusted *p* value≤0.05) when compared to control electroporation with water. CDN: cyclic dinucleotide, CMN: cyclic mononucleotide, CON: cyclic oligonucleotide, LN: linear nucleotide. See also Figure S1 and Table S1.

The nucleotide library was designed to encompass the broadest possible range of candidate signaling molecules, drawing on all known cyclic nucleotides and incorporating recently discovered bacterial cyclic oligonucleotides and their structural variants.^41^^,^^50^^,^^51^^,^^52^ Of note, the library was not limited to known STING agonists. Rather, we extended it to include nucleotides that could potentially signal through alternative pathways, as well as molecules whose signaling capacity in human cells has not previously been established, with the goal of comprehensively profiling nucleotide-mediated signaling beyond the cGAS-STING axis. Thus, we also included linear nucleotides (LNs), given that in humans linear 2′–5′ oligoadenylates function as antiviral second messengers by activating ribonuclease L (RNase L) and inducing translational arrest.^53^^,^^54^^,^^55^ In addition, the linear guanosine tetraphosphate or pentaphosphate (p)ppGpp is a widespread bacterial second messenger involved in stress responses and has recently also been identified in metazoans.^56^^,^^57^ An additional rationale for their inclusion is that linear intermediates are generated during cyclic-nucleotide synthesis or degradation; yet it remains unknown whether these linear species possess signaling capacity in cells. The nucleotide library comprises 78 nucleotides, including 38 CDNs, 9 cyclic oligonucleotides (CONs), 13 cyclic mononucleotides (CMNs) and 18 LNs (Figure 1A; Table S1). CDNs include the natural STING ligand 2′3′-cGAMP, as well as a wide array of cyclic nucleotides that differ in nucleobase composition ranging from purine-containing and pyrimidine-containing species to purine-pyrimidine hybrids. They also encompass large structural diversity, including different combinations of 2′–5′ and 3′–5′ phosphodiester linkages, non-hydrolysable phosphorothioate analogues, and dideoxy cyclic nucleotides. These non-hydrolysable cyclic nucleotide compounds are resistant to hydrolysis by phosphodiesterases such as ENPP1 and are therefore more stable, inducing stronger and prolonged signaling.^26^ CONs include oligonucleotides ranging from three bases (e.g., cyclic tri-AMP) to six bases (e.g., cyclic hexa-AMP), several of which have been identified in bacteria and function in anti-phage defense systems.^41^^,^^50^^,^^51^^,^^52^ CONs also have the potential to signal in human cells as demonstrated by binding to and activation of mammalian reductase controlling NF-κB (RECON) by cyclic AMP-AMP-GMP (cAAG).^41^ CMNs include the well-known nucleotides in humans, 3′,5′-cGMP and 3′,5′-cAMP. cAMP was the first nucleotide second messenger to be discovered and regulates protein kinase A activation downstream of hormone and neurotransmitter signaling.^58^^,^^59^ Additional CMN diversity in the library is covered by nucleotides with 2′,3′ linkage isomers and alternative nucleobases, such as 3′,5′ xanthosine monophosphate (XMP), which has been identified in plants and mice.^60^^,^^61^ LNs in our library consist of adenosine or guanosine chains that range from two to five nucleobases in length. They include cleavage products of bacterial CDNs, such as linear 5′- phosphoguanylyl-(3′-5′)-guanosine (pGpG), a metabolite of c-di-GMP that may also function as an independent signaling molecule.^62^^,^^63^ Overall, our custom-made library comprises a diverse set of nucleotides, including molecules known to signal in human cells serving as controls as well as others whose existence or signaling capacity has not previously been established.

We monitored nucleotide-induced signaling in THP-1 monocytes, which express the STING HAQ (H72 A230 Q293) variant found in ca. 20% of human population.^3^^,^^36^ This variant can be activated by both the metazoan 2′3′-cGAMP and bacterial CDNs with 3′3′ linkage to induce a robust transcriptional response (Figure S1A).^3^^,^^29^^,^^36^ THP-1 monocytes were electroporated with each nucleotide or with water as a control and harvested 6 h later for total transcriptome analysis by RNA-seq (Figure 1B). Untreated cells without electroporation were included as a control to assess the effects of electroporation alone, which induced only minimal changes in gene expression (Figures 1C and 1D). Electroporation ensured rapid delivery of nucleotides into cells minimizing degradation by extracellular phosphodiesterases.^25^^,^^26^^,^^27^ THP-1 monocytes were electroporated with 600 nM of each nucleotide, a concentration at which 2′3′-cGAMP induces robust STING activation and strong expression of *IFNB1* and *CXCL10* (Figure S1A). Principal component analysis (PCA) of RNA-seq transcript counts revealed that all CONs, LNs, CMNs, and a subset of CDNs clustered with control and untreated cells, indicating that these nucleotides did not induce signaling (Figure 1C). The lack of signaling detected for the well-known CMNs cAMP and cGMP in humans may be explained by differences in treatment duration and stimulation conditions, which other gene-expression studies have shown to be critical for inducing a robust response.^64^^,^^65^^,^^66^ In contrast, a distinct group of CDNs clustered separately from control samples, consistent with induction of gene expression changes. Analysis of differentially expressed genes (DEGs), comparing each nucleotide to the control, showed that the endogenous STING ligand 2′3′-cGAMP induced the strongest transcriptional response, with a total of 1196 DEGs (Figure 1D; Table S1). The ten nucleotides inducing the greatest number of DEGs were in fact hydrolysable and non-hydrolysable isomers of cGAMP and c-di-AMP, both of which are established STING agonists. Overall, gene expression changes were induced by 27 CDNs in our library, all of which were composed of purine nucleobases guanosine, adenosine, or inosine and their hydrolysable and non-hydrolysable isomers (Figure S1B). Only 3 DEGs in total were identified for non-CDN nucleotides cyclic tetra-GMP (3′3′3′3′-c-tetra-GGGG) and ApA, which belong to the CONs and LNs class, respectively. Taken together, the results of our nucleotide library screen demonstrate that nucleotide signaling is highly specific, with many compounds failing to elicit any response, while simultaneously revealing that STING displays strong selectivity toward a narrow subset of purine-containing CDNs that activate signaling in THP-1 cells.

### STING activation by distinct CDNs induces a common transcriptional signature

**Figure 2:**
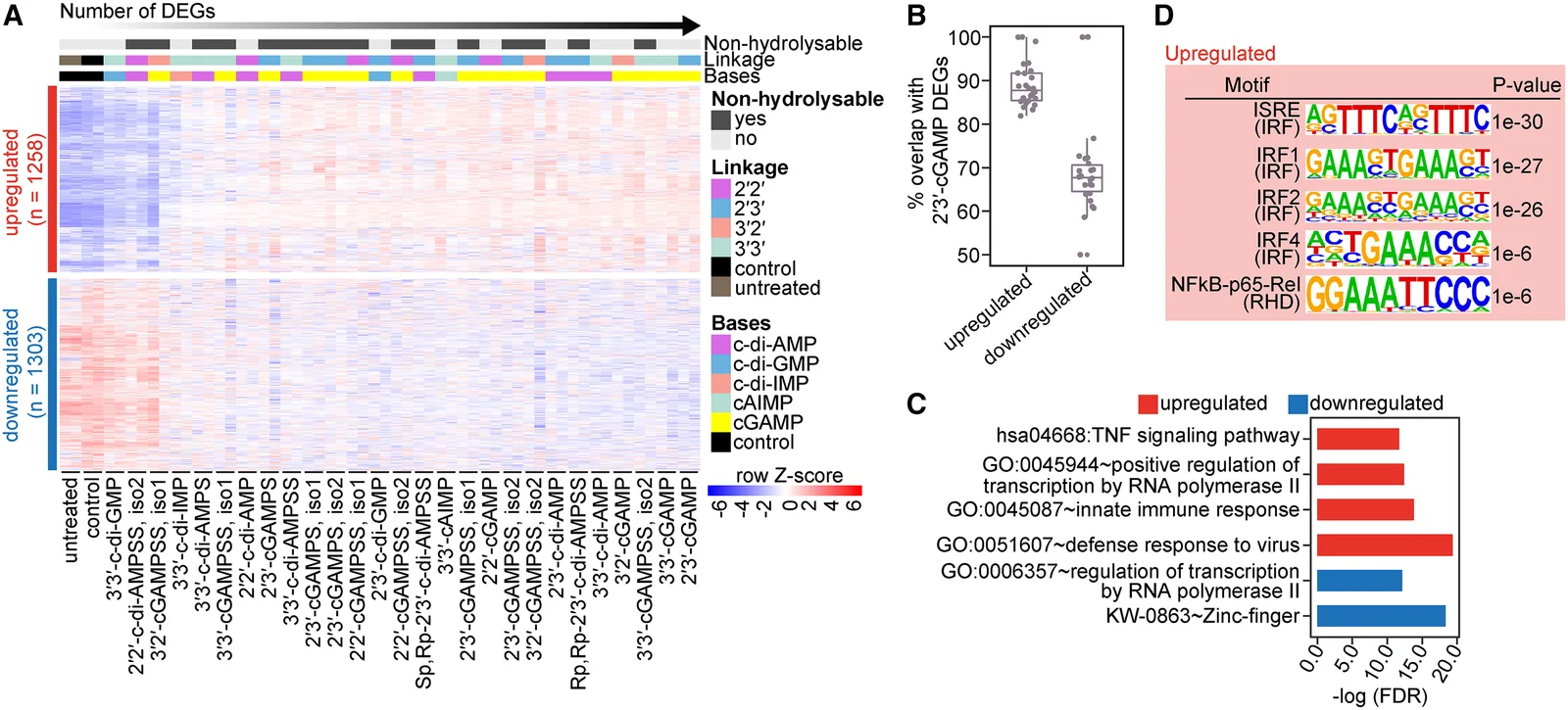
STING activation by distinct CDNs induces a common transcriptional signature (A) Heatmap showing clustering of all DEGs pooled from signaling 27 CDNs, which identified two clusters. Treatments are sorted by the number of DEGs that the nucleotides induced. Normalized transcript counts from variance stabilizing transformation (vst) were used, counts are row scaled. (B) Boxplot showing percentage of upregulated and downregulated DEGs for each signaling CDN (dots) that overlap and were identified as DEGs also for 2′3′-cGAMP. Boxes encompass the twenty-fifth to seventy-fifth percentile changes. Whiskers extend to the tenth and ninetieth percentiles. The central horizontal line indicates the median. (C) Pathway enrichment analysis for the “upregulated” and “downregulated” genes from (A). (D) Transcription factor motif enrichment analysis for the genes from “upregulated” cluster in (A). See also Figure S2.

We next asked whether the responses induced by different CDN isomers were identical, or whether variations in nucleobase composition or phosphodiester linkage resulted in distinct transcriptional outcomes. Several structures of STING bound to different CDNs, including bacterial cGAMP, c-di-AMP, and c-di-GMP, have shown that CDNs can induce distinct STING conformations, suggesting that ligand-specific structural states may influence the magnitude or even type of downstream signaling responses.^4^^,^^49^^,^^67^^,^^68^ In addition, we sought to determine whether individual CDNs elicit responses beyond canonical STING signaling, as exemplified by c-di-AMP in mice, which can also engage the receptor RECON.^48^ Thus, we further analyzed the gene expression signatures induced by the signaling CDNs and compared them to those elicited by 2′3′-cGAMP, which induces exclusively STING-dependent transcriptional changes in THP-1 cells.^69^ Clustering of 2561 unique DEGs, obtained from pooling all DEGs induced by 27 signaling CDNs, revealed two major clusters (Figure 2A). The “upregulated” cluster comprised genes whose expression increased in response to CDN treatment relative to control cells, whereas the “downregulated” cluster contained genes whose expression decreased. All signaling CDNs induced a similar transcriptional signature across both clusters, with variation primarily in magnitude: 3′3′-c-di-GMP induced the weakest changes, while 2′3′-cGAMP triggered the largest number of DEGs and some of the strongest transcriptional responses. This indicates that the CDNs induce responses of varying strengths that closely resemble those triggered by 2′3′-cGAMP, consistent with STING-dependent signaling. Indeed, when analyzing each CDN individually, approximately 90% of upregulated DEGs and 65% of downregulated DEGs were shared with those induced by the endogenous STING agonist 2′3′ -cGAMP (Figure 2B).

To further exclude any STING-independent signaling or transcriptional responses, we analyzed the DEGs induced by all signaling CDNs but not by 2′3′-cGAMP by comparing the overlaps of upregulated and downregulated genes. Pooling the responses of all signaling CDNs uncovered 536 upregulated and 829 downregulated DEGs that were not significantly regulated by 2′3′-cGAMP. (Figure S2A). However, clustering analysis showed that these genes still followed a pattern of regulation similar to that induced by 2′3′-cGAMP, despite not passing the cut-off of log_2_ fold change of [1] and adjusted *p* value of 0.05 thresholds used to define DEGs (Figure S2B). Thus, signaling CDNs converge on a common STING-dependent transcriptional response. The induced genes were strongly enriched for innate immune pathways related to anti-viral defense, including interferon-stimulated genes (ISGs) and NF-κB-driven TNF signaling (Figure 2C). Induction of interferon signaling and the NF-κB pathways is a hallmark of STING activation, which is also reflected in the strong enrichment of ISRE and IRF transcription factor (TF) motifs, as well as NF-κB motifs, among the upregulated DEGs (Figure 2D). Furthermore, both upregulated and downregulated genes were enriched for pathways associated with transcriptional regulation, comprising numerous transcription factors from diverse families (Figure 2C; Figure S2C). The upregulated genes included basic leucine zipper domain superfamily members such as JUNB, FOSL1, FOS, ATF3, CEBPB, and STAT proteins, all of which are well-established regulators of inflammatory responses.^70^^,^^71^ In contrast, the downregulated genes were significantly enriched for Zinc finger (ZNF) transcription factors containing a Krüppel associated box (KRAB) repressor domain (Figure S2C).^72^ Collectively, our analysis identified a set of 27 CDNs that induce a spectrum of STING-dependent gene expression changes, modulating inflammatory and transcriptional pathways.

### Potent STING agonists drive cell death in pancreatic cancer cells

**Figure 3:**
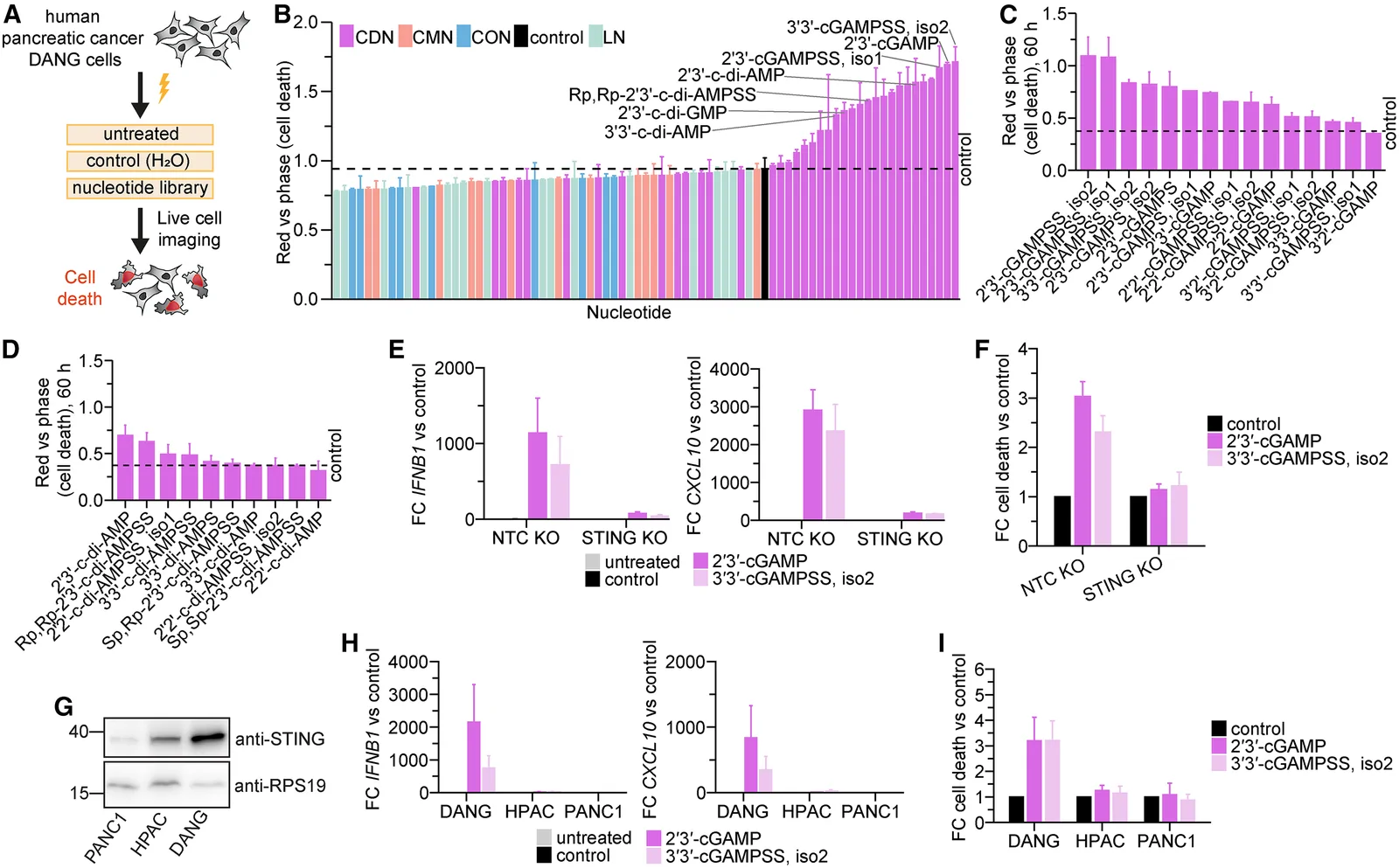
Potent STING agonists drive cell death in pancreatic cancer cells (A) Experimental setup. DANG pancreatic cancer cells were electroporated with 2 μM of each nucleotide from the library in duplicate or water as a control. Cell death was monitored by live cell imaging over 70 h in the presence of red fluorescent cell death dye YOYO-3. (B) Ranking of nucleotides by the level of induced cell death (red vs. phase signal) in DANG cells at 12 h post-treatment. (C and D) Ranking of cGAMP (C) and c-di-AMP (D) isomers from the library for induction of cell death in DANG cells at 60 h post-treatment. (E) RT-qPCR analysis of *IFNB1* and *CXCL10* expression after electroporation of DANG STING KO and NTC KO cells with 2 μM indicated nucleotides or water (control) for 5 h. (F) Analysis of cell death in DANG STING KO and NTC KO cells 24 h after electroporation with water (control), 2 μM 2′3′-cGAMP or 2 μM 3′3′-cGAMPSS, iso2. (G) Western blot analysis showing STING levels in PANC1, HPAC, and DANG cells with anti-RPS19 as a loading control. (H) RT-qPCR analysis of *IFNB1* and *CXCL10* expression after electroporation of DANG, HPAC, and PANC1 cells with 2 μM indicated nucleotides or water (control) for 5 h. (I) Analysis of cell death in DANG, HPAC, and PANC1 cells 24 h after electroporation with water (control), 2 μM 2′3′-cGAMP or 2 μM 3′3′-cGAMPSS, iso2. CDN: cyclic dinucleotide, CMN: cyclic mononucleotide, CON: cyclic oligonucleotide, LN: linear nucleotide. Bars represent means from at least two replicates and error bars represent standard deviation. In (B), (C), (D) dashed line represents levels of red vs. phase (cell death) after control electroporation with water. See also Figure S3.

STING agonists are currently being developed for cancer therapy to induce protective antitumor immunity as well as cancer cell death.^21^^,^^73^^,^^74^^,^^75^^,^^76^ The consequences of STING activation in tumors are multifaceted and shaped by both intratumoral heterogeneity and the surrounding microenvironment.^20^^,^^77^ On the one hand, STING signaling can exert adverse effects by promoting T cell death, thereby generating an immunosuppressive, tumor-supportive microenvironment.^78^^,^^79^^,^^80^^,^^81^ On the other hand, STING activation and downstream interferon signaling can be advantageous by promoting recruitment of effector T cells and eliciting inflammatory anti-tumor responses within the tumor microenvironment and more systemically, as shown in the pancreatic ductal adenocarcinoma (PDAC) models.^82^^,^^83^^,^^84^^,^^85^^,^^86^^,^^87^ Anti-tumor effects of STING signaling in PDAC have been attributed primarily to responses in cancer-surrounding cells, including stromal cells, epithelial cells, and tumor-associated macrophages.^83^^,^^87^^,^^88^^,^^89^ PDAC tumor cells express high levels of STING.^82^^,^^84^^,^^90^ However, the consequences of tumor cell-intrinsic STING signaling have been less studied and have been associated with metabolic reprogramming, cell-cycle arrest, and reduced tumor cell growth.^82^^,^^90^ Given that intrinsic type I interferon signaling in PDAC tumor cells can cause reduced cell growth and cell death, we sought to determine how such responses may be activated downstream of STING by structurally diverse nucleotides, motivating a cell death screen in PDAC cells.^82^^,^^90^^,^^91^^,^^92^^,^^93^ Because STING activation does not necessarily correlate with antitumor activity, we leveraged our nucleotide library together with the distinct response patterns observed in THP-1 cells to define the spectrum of CDNs capable of inducing cell death in pancreatic cancer cells. To preserve the possibility of engaging receptors beyond STING, we included the full nucleotide library, encompassing non-STING agonists, as this represented a new cell line and functional readout. To this end, we electroporated the nucleotide library into the human pancreatic cancer cell line DANG, which carries a STING WT allele^94^ and responds efficiently to interferon stimulation.^82^ Cell death was monitored by time-lapse live-cell imaging using the cell-death dye over 70 h (Figure 3A). Consistent with our transcriptional activation findings in THP-1 cells, induction of cell death in PDAC cells was observed exclusively for purine-containing CDNs (Figure 3B; Figure S3A). The most potent inducers of cell death were the non-hydrolysable isomers of cGAMP and c-di-AMP, as well as 2′3′-c-di-AMP, 2′3′-c-di-GMP and endogenous 2′3′-cGAMP (Figure 3B; Figure S3). The non-hydrolysable CDN isomers induced the highest levels of cell death when measured 60 h after electroporation, consistent with a cellular effect driven by their increased stability and resistance to phosphodiesterase-mediated cleavage (Figures 3C and 3D). To confirm that the observed cell death was STING-dependent, we electroporated 2′3′-cGAMP and non-hydrolysable 3′3′-cGAMP into STING-KO DANG cells, where both compounds failed to induce cell death and transcription of *IFNB1* and *CXCL10* (Figures 3E and 3F; Figure S3B). To further correlate STING signaling capacity with cell death induction, we electroporated 2′3′-cGAMP and non-hydrolysable 3′3′-cGAMP into the PDAC cell lines HPAC and PANC1, which express low STING protein levels, alongside DANG cells, which express high STING levels as previously demonstrated (Figure 3G).^90^ In contrast to DANG cells, neither HPAC nor PANC1 cells showed induction of cell death or upregulation of *IFNB1* and *CXCL10* (Figures 3H and 3I), indicating that CDN-induced cell death in PDAC cells is dependent on STING protein levels and downstream signaling capacity. Electroporation of 2′3′-cGAMP also induced *Ifnb1* expression in mouse pancreatic cancer (mPC) cells (Figure S3C), indicating the potential for beneficial STING signaling across pancreatic cancer models in different organisms. Collectively, our nucleotide-library experiments highlight the remarkable specificity of nucleotide signaling in diverse cell types and demonstrate how the signaling capacity of distinct CDNs can be translated across these cellular contexts and responses.

### STING activation is skewed toward cyclic dinucleotides with 2′3′ phosphodiester linkage

**Figure 4:**
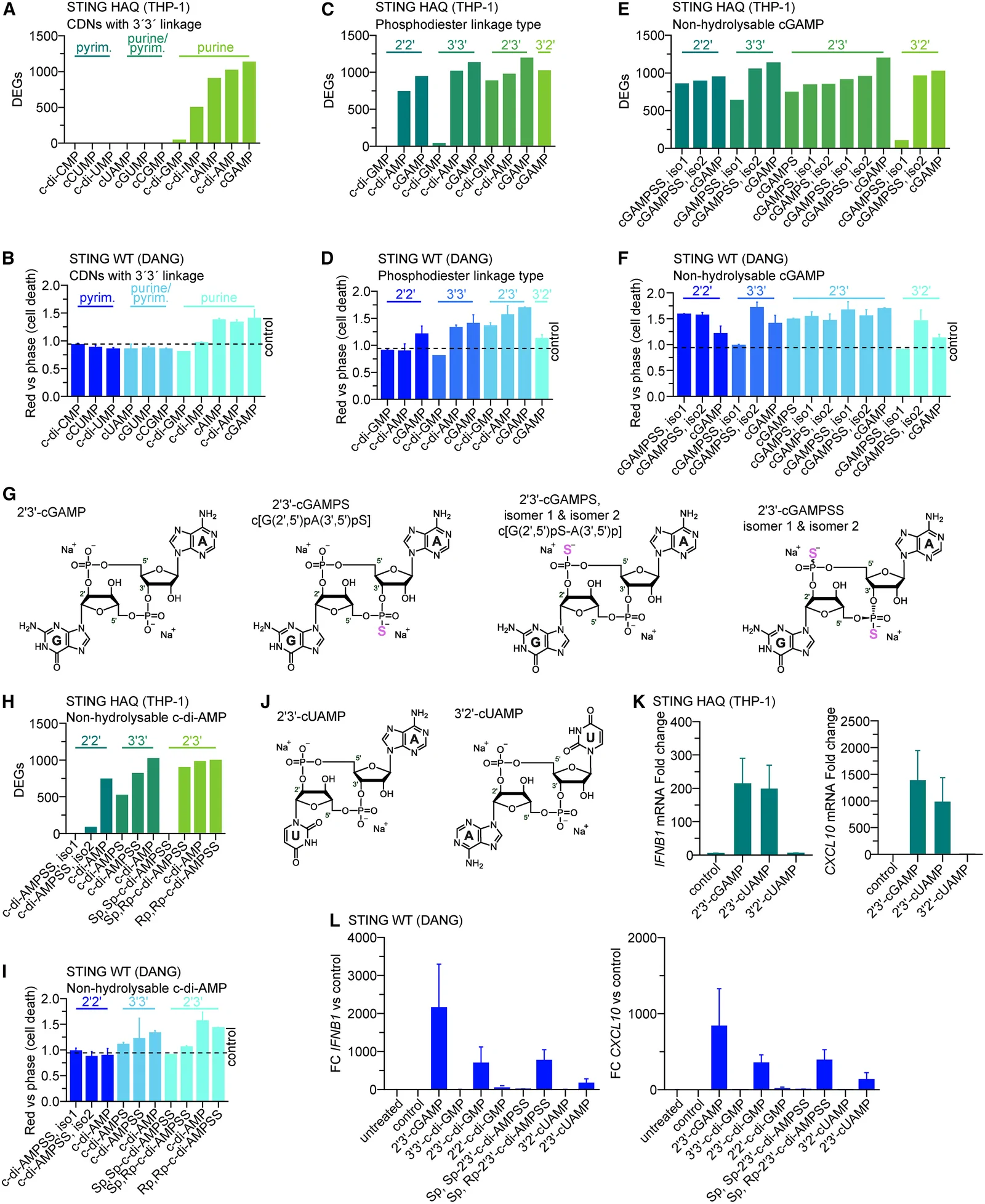
STING activation is skewed towards cyclic dinucleotides with 2′3′ phosphodiester linkage (A and B) Bar plot showing number of DEGs in THP-1 cells (A) and red vs. phase (cell death) in DANG cells (B) for all nucleotides in the library with 3′3′ phosphodiester linkage grouped by purine and/or pyrimidine base composition. (C and D) Bar plot showing number of DEGs in THP-1 cells (C) and red vs. phase (cell death) in DANG cells (D) for all c-di-GMP, c-di-AMP, and cGAMP isomers in the library grouped by the phosphodiester linkage type. (E and F) Bar plot showing number of DEGs in THP-1 cells (E) and red vs. phase (cell death) in DANG cells (F) for all hydrolysable and non-hydrolysable cGAMP isomers in the library. (G) Depiction of different cGAMP isomers containing thiophosphate modifications at different positions. (H and I) Bar plots showing number of DEGs in THP-1 cells (H) and red vs. phase (cell death) in DANG cells (I) for all hydrolysable and non-hydrolysable c-di-AMP isomers in the library. (J) Depiction of 2′3′-cUAMP and 3′2′-cUAMP. (K) RT-qPCR analysis of *IFNB1* and *CXCL10* expression after electroporation of THP-1 monocytes with 600 nM 2′3′-cGAMP, 2′3′-cUAMP, 3′2′-cUAMP, or water (control) for 6 h. (L) RT-qPCR analysis of *IFNB1* and *CXCL10* expression after electroporation of DANG cells with 2 μM indicated nucleotides or water (control) for 5 h. In (B), (D), (F), (I), (K) and (L) bars represent means from at least two replicates and error bars represent standard deviation. In (B), (D), (F) and (I) dashed line represents levels of red vs. phase (cell death) after control electroporation with water. See also Figure S4.

Since no additional signaling was observed from nucleotide compounds other than the CDNs that activate STING and trigger the canonical 2′3′-cGAMP response, albeit with different magnitudes, we next sought to use these findings to identify the shared features that determine STING’s nucleotide activation preferences. Having profiled the complete nucleotide library in both THP-1 cells, which carry the STING HAQ variant,^3^^,^^68^ and DANG cells, which carry the STING WT allele,^94^ our dataset enables a systematic comparison of CDN structural requirements across two naturally occurring human STING variants. Multiple STING SNPs exist in the human population, influencing responsiveness to different CDNs. Both human STING WT and HAQ variants respond to CDNs with a 2′3′ linkage as well as bacterial CDNs with a 3′3′ linkage, whereas the R232H variant is unresponsive to CDNs containing a 3′3′ linkage.^3^^,^^29^^,^^36^ Altogether, our analysis in THP-1 and DANG cells demonstrated STING-dependent signaling for 27 out of 38 CDNs included in our nucleotide library. The overall pattern of active and inactive CDNs was broadly consistent across both cell lines, although a larger number of CDNs induced signaling in THP-1 cells than in DANG cells. Among CDNs with 3′3′ phosphodiester linkages, STING activation was observed exclusively for molecules composed of purine nucleobases in both STING alleles, with pyrimidine-containing CDNs failing to induce signaling (Figures 4A and 4B). We further examined STING's preference for specific phosphodiester linkage types using purine-containing cGAMP, c-di-AMP and c-di-GMP isomers. All 2′2′, 2′3′, 3′3′, or 3′2′ (cGAMP only) linkage isomers of c-di-AMP and cGAMP induced high levels of STING activation (Figures 4C and 4D) with a strong and consistent preference for 2′3′ linkages evident across both STING alleles. Both 2′3′-c-di-GMP and 2′3′-c-di-AMP induced robust signaling comparable to 2′3′-cGAMP (Figures 4C and 4D), whereas 3′3′-c-di-GMP acted as a weak agonist in THP-1 cells but not in DANG cells, and 2′2′-c-di-GMP showed no activity in either. Consistent with this, treatment with 2′3′-c-di-GMP, but not its 3′3′ isomer, induced *IFNB1* and *CXCL10* transcription in DANG cells, further corroborating the preference for 2′3′ phosphodiester linkage (Figure 4L). Notably, in THP-1 cells we identified 2′2′-c-di-AMP as a previously unrecognized STING agonist, thereby expanding the repertoire of known activating CDNs (Figure 4C). The weak activity of 3′3′-c-di-GMP is consistent with earlier reports in mouse and human STING^1^^,^^4^^,^^29^^,^^67^^,^^68^ and is thought to result from its lower affinity, which stabilizes a more open STING conformation and promotes cooperative activation rather than the closed-state polymerization induced by 2′3′-linked ligands and 3′3′-c-di-AMP.^4^^,^^67^^,^^68^

Our nucleotide library includes several phosphorothioate-containing isomers of c-GAMP, c-di-GMP, and c-di-AMP, in which a non-bridging oxygen on one or both phosphates is substituted with a sulfur atom (Figure 4G). This modification confers resistance to phosphodiesterase-mediated cleavage, making these analogues more attractive candidates for immunomodulatory applications.^26^^,^^29^ Among the cGAMP isomers, all non-hydrolysable linkage isomers produced robust STING activation, except for the 3′2′-cGAMPSS isomer 1 and 3′3′-cGAMPSS isomer 1 in DANG cells (Figures 4E and 4F). For the 2′3′-cGAMP analogues, signaling strength was similar whether sulfur was incorporated at both phosphates or restricted to the 2′–5′ or 3′–5′ linkage (Figures 4E, 4F, and 4G), although these phosphorothioate forms generally induced slightly lower activation than unmodified 2′3′-cGAMP in THP-1 cells. In contrast, the behavior of other phosphorothioate-modified CDNs varied by phosphodiester linkage type. In THP-1 cells, while natural 2′2′-c-di-AMP activated STING, introducing sulfur atoms at both phosphates eliminated its activity (Figure 4H). Non-hydrolysable 3′3′-c-di-AMP analogues retained STING activation capacity, albeit at reduced levels compared with unmodified 3′3′-c-di-AMP (Figures 4H and 4I). Conversely, thiophosphate modification of 3′3′-c-di-GMP completely abolished signaling in THP-1 cells (Figure S4A and S4B). The ability of 2′3′-c-di-AMP to induce STING signaling was highly dependent on the stereochemistry of the thiophosphate groups and STING allele. The Rp, Rp dithio-substituted diastereomers triggered strong STING activation, while the Sp, Sp form failed to elicit a response in both THP-1 and DANG cells (Figures 4H, 4I, and L). The mixed Sp, Rp diastereomer displayed intermediate and cell type-dependent activity, inducing strong signaling in THP-1 cells and weaker signaling in DANG cells (Figures 4H, 4I, and 4L). To determine whether these differences in cellular activity reflect differential binding to STING, we performed thermal shift assays with purified recombinant STING ligand-binding domain (LBD). The Sp, Rp form stabilized the STING LBD, whereas the Sp, Sp form did not induce measurable stabilization (Figures S4F and S4G), demonstrating that the capacity for cellular signaling correlates with stabilization of STING’s active conformation. Together, these data indicate that phosphorothioate stereochemistry imposes strict constraints on CDN recognition, with the Rp configuration at one or both positions being required for productive STING engagement. In fact, the Rp, Rp-2′3′-c-di-AMP analogue, also known as ADU-S100 and MIW815, has progressed into clinical trials for advanced and metastatic solid tumors as well as lymphomas, albeit with limited clinical efficacy.^33^^,^^34^ In summary, our analysis of STING responses to 38 distinct CDNs revealed a strong preference for 2′3′ phosphodiester linkage, which outweighed the influence of base composition. Even weaker agonists, such as 3′3′-c-di-GMP, exhibited markedly enhanced signaling when configured with a 2′3′ linkage. In addition, we identified several non-hydrolysable c-di-AMP and cGAMP isomers that induced robust STING activation, highlighting candidates with improved stability and prolonged signaling potential for therapeutic applications.

Recent studies have expanded the diversity of known CDNs as well as cGAS-like enzymes and STING homologs in bacteria, *Drosophila* and metazoans.^37^^,^^38^^,^^39^^,^^40^^,^^41^ Of note, STING homologs in coral *S. pistillata* can be activated not only by 2′3′-cGAMP but also by purine-pyrimidine CDN 2′3′-cUAMP, which contains a 2′3′ phosphodiester linkage^40^ (Figure 4J). Structural studies further demonstrated that 2′3′-cUAMP binds both to *S. pistillata* and human STING and induces a closed conformation similar to that triggered by 2′3′-cGAMP.^4^^,^^40^^,^^67^ The strong preference of human STING for 2′3′-linked CDNs (Figures 4C and 4D), together with the ability of 2′3′-cUAMP to induce a closed, active STING conformation,^40^ suggests that human STING can also be activated by pyrimidine-containing 2′3′-linked CDNs. Indeed, electroporation of THP-1 monocytes with 2′3′-cUAMP resulted in robust induction of *IFNB1* and *CXCL10* expression, with levels comparable to those elicited by 2′3′-cGAMP (Figure 4K). In DANG cells 2′3′-cUAMP-induced signaling was lower when compared to 2′3′-cGAMP (Figure 4L). In contrast, 3′2′-cUAMP and 3′3′-cUAMP failed to induce signaling, indicating a requirement for both the specific pyrimidine base position and the 2′3′ phosphodiester linkage (Figures 4A, B and 4J-L). To confirm the dependence on STING, we generated STING-KO THP-1 cells and verified that the induction of *IFNB1* and *CXCL10* by 2′3′-cUAMP, as well as by the other signaling CDNs in our screen (Figure 2), was abolished in the absence of STING (Figures S4C and S4D). Dose-response analysis of 2′3′-UAMP and 2′3′-cGAMP further showed that 2′3′-cGAMP activated STING more efficiently at lower concentrations, although both CDNs achieved similar activation at higher concentrations (Figure S4E). Taken together, our data reveal that STING can tolerate pyrimidine bases at a specific position for activation and signaling when presented within its preferred 2′3′ phosphodiester linkage.

### Molecular dynamics simulations of STING in complex with 2′3′-cUAMP and 3′2′-cUAMP

Previous structural and biochemical studies have investigated why CDNs containing purine bases and mixed 2′3′ phosphodiester linkages are more potent activators of human STING than those with canonical 3′3′ or 2′2′ linkages, establishing the mixed linkage topology as a key determinant of receptor activation.^4^^,^^49^^,^^67^^,^^68^^,^^95^ Our results extend this picture by showing that even within the preferred 2′3′ linkage context, nucleobase positioning is a critical and previously underappreciated variable: substitution of a purine with a pyrimidine at specific positions can reduce STING activation or lead to its complete abolishment.

**Figure 5:**
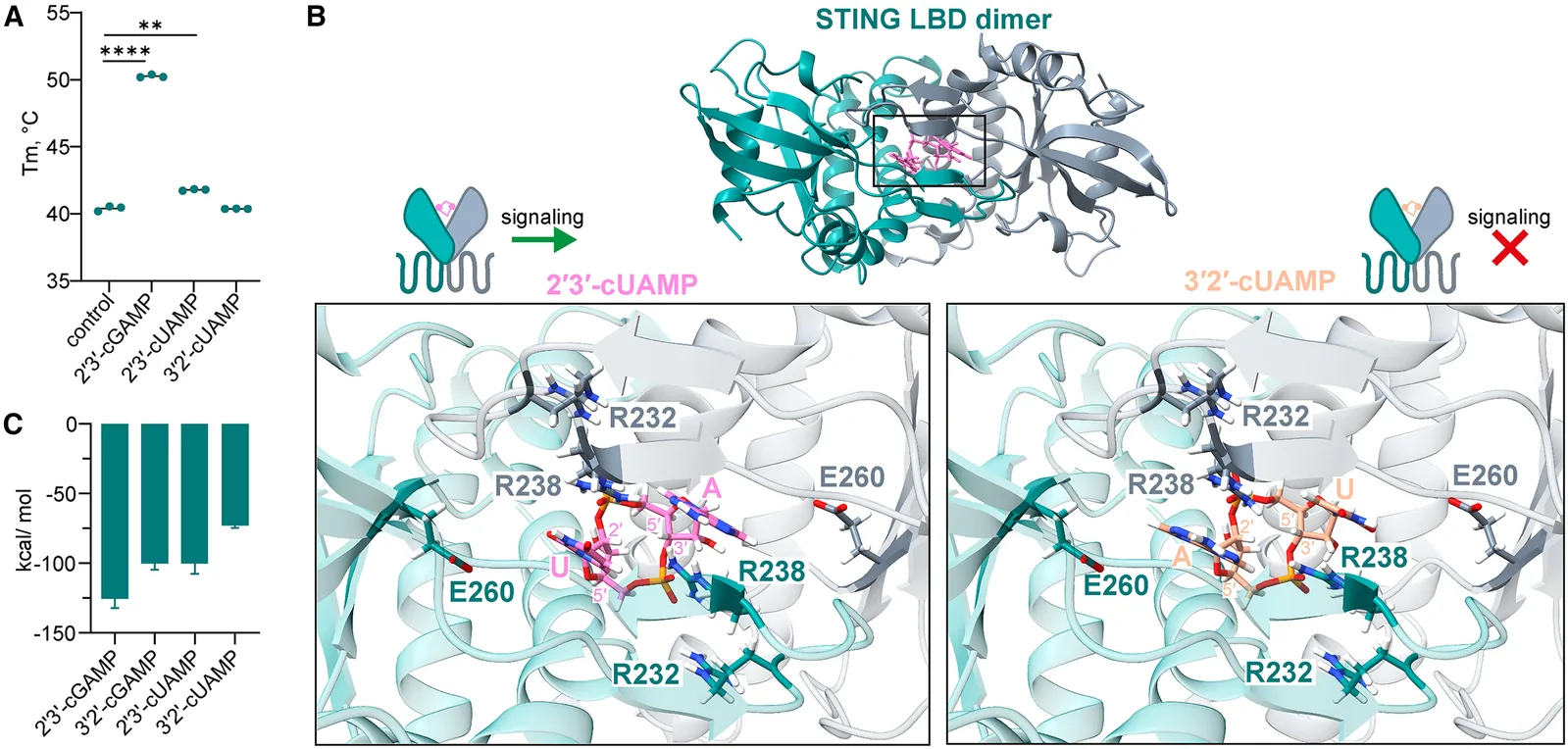
Molecular dynamics simulations of STING in complex with 2′3′-cUAMP and 3′2'-cUAMP (A) Melting temperature analysis of recombinantly purified STING ligand-binding domain (LBD) with water (control), 2′3′-cGAMP, 2′3′-cUAMP, 3′2′-cUAMP by thermal shift assays. Dots: independent replicates, horizontal lines: mean. Paired *t* test ∗∗ *p* ≤ 0.01, ∗∗∗∗ *p* ≤ 0.0001. (B) Top: structure of STING ligand binding domain (LBD) dimer bound to a cyclic dinucleotide. Bottom: close-up view of MD simulation models of STING bound to 2′3′-cUAMP and 3′2′-cUAMP. (C) Calculated mean interaction energies to STING-ligand binding domain (LBD) for the indicated nucleotides using MMGBSA (molecular mechanics generalized born surface area) method. Error bars represent standard errors of the mean. See also Figure S5.

Our cellular data demonstrate that 2′3′-cUAMP activates STING in THP-1 cells carrying the HAQ variant at levels comparable to 2′3′-cGAMP, and similarly activates STING WT, while the isomer 3′2′-cUAMP fails to induce signaling in either STING allele (Figures 4K and 4L; Figures S4D and S4E). To determine whether this functional difference reflects differences in ligand binding and STING dimer stabilization, we performed thermal stability assays with recombinantly purified STING ligand-binding domain (LBD). 2′3′-cUAMP stabilized STING LBD by 1.4°C, whereas 3′2′-cUAMP produced no measurable thermal stabilization, suggesting an inability to stabilize the closed STING dimer conformation required for signaling, consistent with the cellular results (Figure 5A; Figure S5A).

Because available STING crystal and cryo-EM structures^4^^,^^67^^,^^95^^,^^96^ are inherently symmetric and do not fully resolve the discrimination of asymmetric ligands, we performed all-atom molecular dynamics (MD) simulations to characterize how nucleobase positioning differences between 2′3′-cUAMP and 3′2′-cUAMP influence conformational dynamics within the STING ligand-binding pocket. STING dimer complex with 2′3′-cUAMP remained stable throughout several independent simulations with low overall root-mean-square deviation (RMSD) values of the protein (Figure S5B) and of the bound 2′3′-cUAMP ligand (∼1–1.7 Å), with slightly greater conformational fluctuations observed in the 2′3′-cAUMP-bound state (Figure S5B). The simulations identify R232, R238, Y163, Y167, Y240, and E260 as the key coordinating residues in the CDN-binding and lid region, consistent with contacts previously described for 2′3′-cGAMP^4^^,^^68^ (Figure 5B; Figure S5C). Cluster analysis of sampled conformational states indicates that the coordination is not static but transient changes in the interaction pattern of R238 and R232 with the 2′3′-cUAMP as well as 2′3′-cAUMP ligand are possible (illustrated as conformational clusters in Figure S5C). Critically, we observed that E260 occasionally forms direct interactions with R238 (Figure S5C). Binding of negatively charged CDN ligands potentially promotes protonation of E260, disrupting the transient R238–E260 interaction and repositioning R238 to coordinate the phosphate of the incoming CDN. Consistent with a functional role for E260 in ligand coordination, disruption of the R238–E260 hydrogen bond has been independently observed in MD simulations of STING bound to DMXAA, a non-nucleotide murine STING agonist, and mutation of E260I has been shown to improve DMXAA-dependent signaling of human STING,^97^ further supporting the mechanistic importance of this interaction. To further probe the role of E260, we generated E260Q and E260D mutants and observed reduced STING stability during purification of the recombinant protein, suggesting that the E260–R238 interaction plays a stabilizing role in the apo STING dimer in the absence of ligand (Figures S5D and S5E). In 2′3′-cGAMP, the endogenous vertebrate STING ligand, guanine occupies the 2′ position and directly stabilizes E260, resulting in the highest binding affinity.^4^ Notably, in 3′2′-cGAMP, the cGAS-like receptor (cGLR) product in *Drosophila*,^38^^,^^39^ guanine occupies the 3′ position instead, and human STING binds this isomer with lower affinity,^4^ consistent with our cellular data (Figures 4C and 4D). Nonetheless, guanine stabilizes E260 more effectively than uracil regardless of its position in the CDN scaffold. In 2′3′-cUAMP, uracil occupies the 2′ position in place of guanine and, being less bulky, provides additional space for R238 to retain interaction with E260, resulting in partial dissociation of R238 from the CDN phosphate. This reduces binding stability relative to 2′3′-cGAMP but remains compatible with productive STING activation. In 3′2′-cUAMP, uracil instead occupies the 3′ position in place of adenine. At this position the nucleobase sits directly on the sugar ring and is more geometrically constrained than at the 2′ position, which is slightly displaced (Figure 5B). Calculations of the mean STING interaction with 2′3′-cGAMP, 3′2′-cGAMP, 2′3′-cUAMP, and 3′2′-cUAMP are in line with these findings, showing that the calculated binding energies correspond to the relative STING signaling capacity of each CDN observed in cells (Figures 5C and 4K, and 4L).

## Discussion

As the diversity of nucleotide second messengers continues to expand, particularly in bacteria,^41^^,^^50^ it has become increasingly important to determine whether these molecules can elicit signaling in human cells either through alternative pathways or via STING. Given that human STING responds to both endogenous metazoan 2′3′-cGAMP and selected bacterial CDNs,^1^^,^^2^^,^^3^^,^^42^^,^^43^^,^^44^^,^^45^ we systematically evaluated whether other nucleotide second messengers can activate STING in human cells. Previous nucleotide screens investigating STING signaling have used diverse human and mouse cell types expressing different STING variants and have monitored distinct cellular responses, making it difficult to analyze and compare STING activation across studies.^29^^,^^98^^,^^99^^,^^100^^,^^101^ Moreover, purine-containing CDNs have traditionally been the primary focus when analyzing STING agonists. Using our comprehensive nucleotide library, we evaluated the ability of diverse CDNs from various organisms, including those containing pyrimidine bases as well as other cyclic nucleotides such as cyclic mononucleotides and oligonucleotides, to activate endogenous STING in THP-1 monocyte cells and pancreatic cancer cells. We observed a striking specificity of nucleotide responses, identifying a distinct set of nucleotides that induced signaling (27 CDNs in total) and others that failed to do so, underscoring the remarkable selectivity that cyclic nucleotide receptors exhibit toward their respective ligands. Overall, our findings support the idea that additional nucleotide receptors beyond STING, with similarly high specificity, may be uncovered by monitoring nucleotide-mediated signaling in other cell types and experimental contexts.

In line with our data, the purine-containing CDNs were reported to be the most potent STING agonists in other nucleotide screens with different cellular models and readouts: (1) in suppression of virus infection with SARS-CoV-2, chikungunya virus (CHIKV), West Nile virus (WNV), and Zika virus (ZIKV) in human fibroblasts (HFF-1) and Calu-3 cells^98^^,^^99^; (2) in induction of IRF3 reporter activation in the mouse macrophage cell line analyzing 3′3′ CDNs composed of A, G, C, and U nucleobases^100^ and (3) in interferon reporter activation in HEK293T cells expressing the major STING variants and analyzing inosine-containing CDNs in combination with A, G, C, and U nucleobases, across 2′3′, 2′2′, and 3′3′ linkages.^101^ Analysis of pyrimidine-containing nucleotides has focused primarily on CDNs with 3′3′ linkages, which we show are not STING’s preferred linkage type. Indeed, in the context of 2′3′ and 2′2′ linkages, robust STING activation was observed for the mixed purine-pyrimidine CDNs 2′2′-cUIMP and 2′3′-cUIMP.^101^ This study, together with our observation that human STING is activated by the pyrimidine-containing CDN 2′3′-cUAMP, challenges the previously proposed strict preference for purine-containing CDNs. It indicates that combinations of different linkage types broaden the repertoire of CDNs capable of activating STING to include pyrimidine-containing nucleotides. In addition, our results extend this picture by demonstrating that even within the preferred 2′3′ linkage context, nucleobase positioning is a critical and previously underappreciated variable: pyrimidine substitution at specific positions reduces STING activation or abolishes it entirely. Signaling by pyrimidine-containing CDNs introduces an exciting dimension to the STING research field, particularly regarding the development of non-hydrolysable analogues for therapeutic use and the characterization of their cellular transport and degradation.^22^^,^^23^^,^^24^^,^^25^^,^^26^^,^^27^

Many of the CDNs and STING activators in our library belong to a rapidly expanding list of bacterial CDNs that play important roles in bacterial homeostasis, virulence and anti-phage defense.^102^^,^^103^ STING activation by bacterial CDNs is critical for mounting protective innate immune responses during infection of Gram-positive bacteria *Listeria* and Gram-negative *Chlamydia trachomatis*.^45^^,^^104^^,^^105^ These findings suggest that sensing bacterial CDNs or their degradation products represents a valuable strategy for detecting invading bacteria, either through STING or additional nucleotide-sensing receptors.^106^^,^^107^ For instance, STING activation may be particularly relevant in the gut, for sensing multiple microbiota-derived CDNs, in addition to detecting pathogenic disruption of the epithelial barrier.^108^^,^^109^^,^^110^ Our investigation is limited to the THP-1 monocyte and PDAC cell lines, and we cannot exclude the possibility that these nucleotides may signal in other human cell types, particularly if alternative receptors, other than STING, are expressed differentially across tissues and not present in THP-1s or PDACs.

Our analysis reveals a broad set of STING agonists that induce activation to varying degrees, which likely corresponds to differences in ligand binding strength and their ability to drive STING dimer closure. High-affinity ligands such as 2′3′-cGAMP and 3′3′-c-di-AMP engage deeply within the binding pocket and induce a closed dimer conformation. The 2′3′ linkage imposes unique structural constraints that promote this closed conformation leading to STING polymerization and downstream activation.^4^^,^^40^^,^^67^^,^^68^ In contrast, the earliest described bacterial STING agonist, 3′3-c-di-GMP, functions as a weaker activator both in cells and *in vitro*, driving a more open, apo-like STING conformation and an alternative cooperative mode of activation.^4^^,^^29^^,^^67^^,^^98^^,^^99^ Downstream signaling, in particular, is a critical aspect to consider in the development of effective STING agonists. Despite promising preclinical studies demonstrating the induction of antitumor immunity, most developed STING agonists have failed to translate these effects in clinical trials.^19^^,^^21^^,^^111^ For instance, the non-hydrolysable 2′3′-*c*-di-AMPSS (Rp,Rp) is under clinical development for the treatment of metastatic, solid tumors or lymphomas, but has so far shown limited efficacy in clinical trials.^33^^,^^34^ Additional strategies are being explored^18^^,^^112^ and may include gene therapies to deliver enzymes in cells for synthesis of STING-activating ligands^111^ or mRNA encoding constitutively active STING.^113^ Other approaches involve modifications of STING agonists including altering the position of thiophosphate substitution,^114^^,^^115^ modifying phosphodiester linkages,^116^ incorporating locked nucleic acids^117^ or dideoxy derivatives,^118^ designing sugar-modified analogues,^119^ inosine-containing CDNs,^101^^,^^120^ non-nucleotide based agonists,^121^^,^^122^^,^^123^ intermetallic nanoparticles encapsulating CDNs.^124^ Collectively, these findings demonstrate that the STING ligand-binding pocket accommodates a broad range of hydrolysable, non-hydrolysable, and chemically modified CDNs, providing multiple avenues for the development of more effective STING agonists. While our screen did not reveal nucleotide signaling pathways beyond STING under the tested conditions, this likely reflects the selectivity of CDN receptor recognition rather than an absence of such pathways in humans. The diversity of uncharacterized nucleotidyltransferases and enzymes that synthesize nucleotide second messengers represents a rich area for future exploration, and defining their products and cognate receptors will be an important next step toward a comprehensive understanding of nucleotide-based innate immune signaling.

### Limitations of the study

We provide a systematic functional comparison of a structurally diverse nucleotide library that we anticipate will serve as a resource for the STING agonist and innate immunity community. A limitation of our study is that no STING-independent signaling was detected for any compound in our library. Several factors may account for this, including the absence of relevant receptors in the selected cell types or a structural mismatch between the tested compounds and their true endogenous counterparts. Future studies employing additional cell lines and endogenous nucleotide variants will be important to fully define the scope of CDN-based signaling beyond STING. Furthermore, electroporation was chosen to efficiently deliver nucleotides inside the cells for analysis of intracellular signaling as many nucleotides in our library may lack dedicated cellular importers and to avoid their degradation by extracellular phosphodiesterases. Electroporation does not allow to study intercellular transport, uptake and signaling of nucleotides and may have induced some cellular events, which affect signaling by different nucleotides. In addition, the primary cell lines used, THP-1 monocytes expressing the HAQ allelic variant and DANG pancreatic cancer cells expressing the WT allelic variant, may not capture the full diversity of human STING responses across allelic backgrounds and tissue contexts. Functional screening in additional cell lines carrying other STING alleles will be important to determine whether the observed linkage and nucleobase preferences are general properties in all human STING alleles.

## Resource availability

### Lead contact

Further information and request for data and resources should be directed to, and will be fulfilled by, the lead contact, Carina C. de Oliveira Mann (carina.mann@tum.de).

### Materials availability

All unique/stable reagents generated in this study are available from the lead contact upon reasonable request.

### Data and code availability

• Raw sequencing data have been uploaded to European Nucleotide Archive (ENA): PRJEB102862. The MD simulation trajectories and datasets for plots, unprocessed Western Blot, gel and microscopy images from this study were deposited on Mendeley: https://doi.org/10.17632/fzjk6ttfmc.1. These data are publicly available as of the date of publication.
• This paper does not report original code.
• Any additional information required to reanalyze the data reported in this paper is available from the lead contact upon request.

## Acknowledgments

We thank all the members of the NTase laboratory for helpful comments and discussion. We thank Prof. Andreas Pichlmair (TUM) for support with the live cell imaging platform Incucyte. We thank Frank Schwede (Biolog LSI) for his assistance in conceptualizing the nucleotide library. The work was funded by the 10.13039/501100001659German Research Foundation Emmy Noether Program 458004906 (C.C.d.O.M.), 10.13039/501100000781European Research Council ERC-StG-2023 101117085 (C.C.d.O.M.), a FEBS Excellence Award (C.C.d.O.M.) and Cluster for Nucleic Acid Sciences and Technologies—NUCLEATE, to C.C.d.O.M. and R.R. I.B. is supported by a 10.13039/501100001659DFG Walter Benjamin Fellowship.

## Author contributions

The project was conceived and experiments designed by I.B. and C.C.d.O.M. I.B. performed RNA-seq experiments and data analysis as well as live-cell imaging experiments. M.R.S. performed RT-qPCR analysis. RNA-seq library preparation and sequencing were performed by R.Ö. and R.R. Y.H. generated DANG STING KO cell line. S.V.E. performed STING purification and thermal shift assays. P.K.Q. and M.Z. performed MD simulations. The manuscript was written by I.B., and C.C.d.O.M., and all authors contributed to editing the manuscript and support the conclusions.

## Declaration of interests

The authors declare no competing interests.

## STAR★Methods

### Key resources table

**Table:** 

REAGENT or RESOURCE	SOURCE	IDENTIFIER
**Antibodies**		

rabbit monoclonal anti-STING	Cell Signaling	Cat#13647; RRID: AB_2732796
rabbit polyclonal anti-RPS19	Thermo Fisher Scientific	Cat#A304-002 A; RRID: AB_2620351

**Bacterial and virus strains**		

*E. coli* Rosetta	Expression Systems	N/A
*E. coli* STBL3	Andreas Pichlmair	N/A
*E. coli* DH5alpha	Andreas Pichlmair	N/A

**Chemicals, peptides, and recombinant proteins**		

Nucleotide library	See Table S1	N/A
PEI	Polysciences	Cat#24765
cOmplete protease inhibitor, EDTA free	Roche	Cat#04693132001
YOYO-3	Thermo Fisher Scientific	Cat#Y3606
SYPRO Orange	Sigma-Aldrich	Cat#S5692-50UL
Maxima RT Polymerase	Thermo Fisher Scientific	Cat# EP0753
Ni-NTA agarose	Macherey Nagel	Cat#745400.100

**Critical commercial assays**		

LIVE/DEAD Cell Imaging Kit (488/570)	Thermo Fisher Scientific	Cat#R37601
PrimerScript RT (gDNA Eraser)	Takara	Cat#RR047B
Neon Transfection kit	Thermo Fisher Scientific	Cat#MPK1096; Cat# MPK10096
NucleoSpin RNA Plus kit	Macherey Nagel	Cat#740984.250
PowerUp™ SYBR™ Green Master Mix	Applied Biosystems Thermofisher	Car#A25776
Ultra II FS kit	NEB	Cat# E7805L
SuperSignal West Femto kit	Thermo Fisher Scientific	Cat#34096

**Deposited data**		

RNA-seq	This paper	ENA: PRJEB102862
Human STING MD Simulation	This paper	Mendeley: https://doi.org/10.17632/fzjk6ttfmc.1
Unprocessed WB, gel and microscopy data	This paper	Mendeley: https://doi.org/10.17632/fzjk6ttfmc.1

**Experimental models: Cell lines**		

THP-1	Veit Hornung	N/A
HEK293T	ATCC	Cat#CRL-11268
mPC 53074	Roland Rad	N/A
THP-1 NTC KO	This paper	N/A
THP-1 STING KO	This paper	N/A
HPAC	Roland Rad	RRID:CVCL_3517
PANC1	Roland Rad	RRID:CVCL_0480
DANG	Roland Rad	RRID:CVCL_0243
DANG NTC KO	This paper	N/A
DANG STING KO	This paper	N/A

**Oligonucleotides**		

STING KO gRNA1: CGGTCGG CCCGCCCTTCACT	This paper	N/A
STING KO gRNA2: GGGAATT TCAACGTGGCCCA	This paper	N/A
NTC KO gRNA1: CGACGGAG GCTAAGCGTCGCAA	This paper	N/A
NTC KO gRNA2: CGCGCTT CCGCGGCCCGTTCAA	This paper	N/A
Primers for RT-qPCR, see Table S2	This paper	N/A

**Recombinant DNA**		

pLentiCRISPRv2 (puromycin)	Sanjana et al.^125^	Addgene Plasmid #52961
pMD2-VSVG	Andreas Pichlmair^126^	N/A
pCMV-Gag-Pol	Andreas Pichlmair^126^	N/A
Human STING 139–379 pET28M-SUMO	This paper	N/A
Human STING 139–379 E260Q pET28M-SUMO	This paper	N/A
Human STING 139–379 E260D pET28M-SUMO	This paper	N/A

**Software and algorithms**		

Amber22	Case at al.^127^	https://ambermd.org/
AlphaFold3	Abramson et al.^128^	https://alphafoldserver.com
Drop-seq pipeline (v1.12)	Macosko et al.^129^	https://cumulus.readthedocs.io/en/latest/drop_seq.html
Fiji (ImageJ 2.9.0)	Schindelin et al.^130^	https://imagej.net/software/fiji/
Incucyte S3 software	Sartorius	N/A
DESeq2 1.42.1	Love at al.^131^	https://bioconductor.org/packages/release/bioc/html/DESeq2.html
limma 3.58.1	Ritchie et al.^132^	https://bioconductor.org/packages/release/bioc/html/limma.html
ggplot2 3.4.4	https://ggplot2.tidyverse.org	https://cran.r-project.org/web/packages/ggplot2/index.html
RStudio 2024.09.1 + 394	Posit	https://posit.co/download/rstudio-desktop/
HOMER	Heinz et al.^133^	http://homer.ucsd.edu/homer/
SnapGene	SnapGene	https://www.snapgene.com
UCSF ChimeraX 1.9rc20241121025	Meng et al.^134^	https://www.cgl.ucsf.edu/chimerax/index.html
Prism 10	GraphPad Software	https://www.graphpad.com

### Experimental model and study participant details

#### *Escherichia coli* strains

*E. coli* DH5alpha was used for cloning. *E. coli* STBL3 was used for cloning for plasmids used for lentivirus generation. *E. coli* Rosetta was used for recombinant protein expression.

#### Mammalian cell lines

THP-1 wild type, THP-1 NTC KO (this study) and THP-1 STING KO (this study) cells were cultured in suspension in RPMI supplemented with 10% FBS at 37°C in 5% CO_2_ and passaged at dilution 1:10. THP-1 cells were a kind gift from Prof. Veit Hornung (Gene Center Munich). Pancreatic cancer (PDAC) cell lines DANG, HPAC and PANC1 and mouse pancreatic cancer (mPC) 53704 were a kind gift from Prof. Roland Rad (TUM, Munich). PDAC cell lines DANG, HPAC and PANC1, DANG STING KO (this study), DANG NTC KO (this study) and HEK293T cells (ATCC Cat#CRL-11268) were cultured in DMEM supplemented with 10% FBS at 37°C in 5% CO_2_ and passaged at a dilution 1:5 - 1:10 by washing with PBS and detached with 0.25% trypsin. All cell lines were regularly tested negative for mycoplasma contamination using TaKaRA PCR Mycoplasma Detection Set (TaKaRa).

### Method details

#### Protein expression and purification

Human STING C-terminal ligand binding domain (amino acids 139–379) wild-type, E260Q and E260D mutants were cloned into a pET-SUMO vector with N-terminal His-tag. For the expression plasmids were transformed into *E. coli* Rosetta and a 40 mL pre-culture in LB medium supplemented with Kanamycin (50 mg/L) and Chloramphenicol (34 mg/L) was grown at 37 °C, shaking overnight. The next day 10 mL of the pre-culture was used to inoculate 1 L of TB medium supplemented with Kanamycin (50 mg/L) and Chloramphenicol (34 mg/L) and was grown to OD600 = 0.9. The expression was induced by adding IPTG (1 mM) and the culture was further grown overnight at 18°C shaking. The cell pellet was harvested by centrifugation and flash frozen in liquid nitrogen. For purification cell pellet was thawed on ice and resuspended in lysis buffer (20 mM HEPES pH 7.5, 400 mM NaCl, 10% Glycerol, 30 mM Imidazole, 1× protease inhibitor (100 mM PMSF, 200 mM benzamidine, 200 μM pepstatin A, 60 μM leupeptin), 1 mM β-mercaptoethanol) and lysed by sonication. The soluble fraction was cleared by centrifugation and used for purification by Ni-affinity chromatography using Ni-NTA agarose (Macherey Nagel) with wash buffer (20 mM HEPES pH 7.5, 1 M NaCl, 10% Glycerol, 30 mM Imidazole, 1× protease inhibitor (100 mM PMSF, 200 mM benzamidine, 200 μM pepstatin A, 60 μM leupeptin), 1 mM β-mercaptoethanol) and elution buffer (20 mM HEPES pH 7.5, 400 mM NaCl, 10% Glycerol, 300 mM Imidazole, 1× protease inhibitor (100 mM PMSF, 200 mM benzamidine, 200 μM pepstatin A, 60 μM leupeptin), 1 mM β-mercaptoethanol). Afterwards, the His-SUMO tag was removed by proteolytic cleavage with SenP2 protease (1:250) (in-house production) during dialysis overnight in buffer containing 20 mM HEPES pH 7.5, 250 mM NaCl, 1 mM TCEP. Proteins were further purified by size-exclusion chromatography with a HiLoad 16/600 Superdex 75 pg (Cytiva) column in 20 mM HEPES pH 7.5, 250 mM NaCl, 1 mM TCEP buffer. Purified STING constructs were concentrated, flash frozen in liquid nitrogen and stored at −80°C.

#### *In vitro* thermal shift assays

For the analysis of STING C-terminal ligand binding domain (amino acids 139–379) wild-type with different CDNs protein was used at a final concentration of 5 μM with a 4× excess of nucleotides and SYPRO Orange dye (Sigma-Aldrich) at a final concentration of 2.5×. The experiment was performed in a buffer with the final concentration of 80 mM NaCl, 0.2 mM TCEP, 0.6 mM DTT and 20 mM HEPES pH 7.5. Samples were measured with Gain 2 and a melting curve ranging from 25°C to 95°C using qTOWER Iris qPCR device (Analytik Jena). Data were analyzed using qTOWER Iris software (Analytik Jena).

For the analysis of STING C-terminal ligand binding domain (amino acids 139–379) wild-type, E260Q and E260Q protein was used at a final concentration of 5 μM protein per well and SYPRO Orange (Sigma-Aldrich) at a final concentration of 1×. The experiment was performed in a buffer with the final concentration of 55 mM NaCl, 0.7 mM TCEP, 6% glycerol and 20 mM HEPES pH 7.5. Samples were measured with Gain 2 and a melting curve ranging from 5°C to 75°C using qTOWER Iris qPCR device (Analytik Jena). Data were analyzed using qTOWER Iris software (Analytik Jena).

#### Molecular dynamics simulations

The structure of the human STING dimer in complex with the 2′3′-cUAMP (pdb: 8gjz) served as a starting and reference structure. Missing residues in the crystal structure were added using AlphaFold3.^128^ Structural models of the complex with 2′3′cAUMP and other CDNs were generated after the best superposition of the sugar phosphate backbone on the reference structure and energy minimization to remove any residual sterical strain. All energy minimization and MD simulations were performed using the Amber22 package.^127^ The parm19SB force field was used for the protein and the Gaff force field^135^ was used to model the ligands. The STING complexes were solvated in octahedral boxes with explicit OPC water molecules^136^ keeping a minimum distance of 12 Å between protein atoms and box boundaries. The ion concentration was adjusted to 0.15 M with sodium and chloride ions. The simulation systems were energy minimized (5000 steps) after solvation followed by heating up to 300 K in steps of 100 K with positional restraints on all heavy atoms of the proteins. Subsequently, positional restraints were gradually removed from an initial 10 kcal・mol^−1^・Å^−2^ to 0.5 kcal・mol^−1^・Å^−2^ within 0.5 ns. In each case 3 independent production simulations of 50 ns with different random seeds for a Langevin thermostat (300 K) were performed at a temperature of 300 K and a pressure of 1 bar. The hydrogen mass repartition option of Amber was used to allow for a time step of 4 fs.^137^ The mean interaction energy between ligand and STING was calculated for each trajectory using the MMGBSA method as implemented in the Amber22 package (igb = 5 option) averaged over 250 trajectory frames. Averages and errors of the mean were obtained from 3 independent runs per bound CDN.

#### Generation of cell lines

Two different gRNA sequences for STING and non-targeting control (NTC) knockout were cloned in pLentiCRISPRv2 vector (Addgene #52961^125^) with puromycin resistance. Guide RNA sequences were STING gRNA1 CGGTCGGCCCGCCCTTCACT, STING gRNA2 GGGAATTTCAACGTGGCCCA, NTC gRNA1 CGACGGAGGCTAAGCGTCGCAA, NTC gRNA2 CGCGCTTCCGCGGCCCGTTCAA. Primers encoding target guide RNA sequences were annealed and used for ligation with linearized vector from restriction digest with BsmbI enzyme (NEB). Successful cloning was verified by sequencing and target vectors were further used for lentivirus generation.

Lentiviral transductions were used to generate THP-1 STING KO, THP-1 NTC KO, DANG STING KO and DANG NTC KO cell lines. For knockout generation lentiviral particles were generated by transfecting HEK293T cells using PEI transfection reagent (Polysciences) with pCMV-Gag-Pol^126^ and pMD2-VSVG^126^ packaging plasmids together with pLentiCRISPRv2 vector with puromycin resistance encoding *S. pyogenes* CRISPR-Cas9 and two different gRNA sequences. pCMV-Gag-Pol and pMD2-VSVG plasmids were kindly provided by Prof. Andreas Pichlmair (TUM, Munich). Lentiviral particles were harvested 48 h post transfection and used to transduce THP-1 and DANG cells followed by selection with 1.5 μg/mL and 1.0 μg/mL puromycin one day post infection, respectively. Selection was terminated once there were no more viable control untransduced cells with puromycin selection. Successful KO was validated by WB analysis.

#### Western blot analysis

For validation of STING KO in THP-1 and DANG cells, cells were lysed in NP-40 lysis buffer (50 mM Tris-HCl pH 7.5, 150 mM NaCl, 1% NP-40, 5 mM EDTA) supplemented with 1× cOmplete protease inhibitor (Sigma-Aldrich) for 20-30min on ice. Soluble fraction was separated by centrifugation for 10min, 21′000xg, 4°C, mixed with 1× Laemmli sample buffer and boiled for 5-10min at 95°C. Protein was resolved by 12% SDS-PAGE and transferred to 0.45 μm PVDF membrane. Membranes were blocked in 5% non-fat dry milk, 0.1% Tween 20 in PBS and incubated with the following primary antibodies: anti-STING (Cell Signaling, #13647, 1:1000 dilution), anti-RPS19 (Thermo Fisher, #A304-002 A, 1:2000 dilution). Afterwards membranes were probed with HRP-conjugated secondary antibody goat anti-rabbit IgG (Dako, P0448). Immunoblots with HRP signal were developed with the SuperSignal West Femto kit (Thermo Fisher Scientific) and imaged with the Bio-Rad ChemiDoc Imaging System or Vilber Fusion FX6 Edge V0.7 Imaging System.

#### Electroporation of nucleotides

The nucleotide library consists of compounds synthesized and quality-controlled by Biolog LSI. All nucleotides are commercially available from Biolog LSI, and their corresponding catalog numbers are provided in Table S1.

Electroporation was performed with Neon Transfection kit (Thermo Fisher Scientific) following manufacturer’s instructions. THP-1 cells were collected and washed with PBS and resuspended in Buffer R to a final density of 5 × 10^6^ cells/mL with 600 nM of nucleotides if not indicated otherwise. For electroporation 5 × 10^5^ cells were used with 100 μL Neon tip and electroporated with pulse voltage 1400 V, pulse width 20 m s and pulse number 2 in 3 mL E2 Electrolyte Buffer. Afterwards cells were transferred to 1 mL pre-warmed RPMI, 10% FBS media in a 24 well plate and incubated at 37°C in 5% CO_2_ for 6 h until harvest.

For cell death analysis with the nucleotide library DANG cells were detached, washed with PBS and resuspended in Buffer R to a final density of 1.5 × 10^6^ cells/mL with 2 μM of nucleotides. Cells were electroporated with 10 μL tip with pulse voltage 1400 V, pulse width 20 m s and pulse number 1 in 3 mL E Electrolyte Buffer. Afterwards cells were transferred to a well in a 96-well plate filled with DMEM, 10% FBS and 250 nM YOYO-3 (Thermo Fisher Scientific) cell death dye. Cell death was monitored using live cell imaging system Incucyte S3 (Sartorius) over 70 h with scans every 2 h. Cell death was quantified as red signal vs. phase.

For electroporation of mPC 53074 cells, cells were washed with PBS and resuspended in Buffer R to a final density of 6 × 10^6^ cells/mL with 1 μM of nucleotides. Cells were electroporated with 100 μL tip with pulse voltage 1400 V, pulse width 20 m s and pulse number 2 in 3 mL E2 Electrolyte Buffer. Afterwards cells were transferred to a well in a 12-well plate filled with 1 mL DMEM, 10% FBS for 6 h until harvest.

DANG, HPAC, PANC1, DANG NTC KO and DANG STING KO cells for analysis of cell death and *IFNB1* and *CXCL10* expression levels were electroporated using Lonza 4D-Nucleofector X-unit (Lonza). Cells were washed with PBS and resuspended in electroporation buffer (120 mM Na_2_HPO_4_/NaH_2_PO_4_ (pH 7.2), 5 mM KCl, 15 mM MgCl_2_) to a final density 10 × 10^6^ cells/mL with 2 μM of nucleotides. Cells were electroporated in 20 μL-well strips with pulse code EN150 (DANG, HPAC) and CM137 (PANC1). For RT-qPCR analysis cells from one well were seeded in a 12-well plate filled with 1 mL DMEM, 10% FBS for 5 h until harvest. For cell death analysis one-eighth of cells from one well in 20 μL-well strips were seeded in 100 μL DMEM, 10% FBS in one well in a 96-well plate for 24 h until cell death analysis using LIVE/DEAD Cell Imaging Kit (488/570) (Thermo Fisher Scientific) following manufacturers’ instructions. For cell death analysis cells were imaged using EVOS M500 fluorescence microscope and cell death was quantified as area of green (live cells) versus red (dead cells) fluorescence using Fiji (ImageJ 2.9.0) software.^130^

#### RT-qPCR analysis

Total RNA was extracted using the NucleoSpin RNA Plus kit (Macherey-Nagel) according to the manufacturers’ protocol. Total RNA was used for reverse transcription with PrimeScript RT reagent Kit with gDNA Eraser (TaKaRa) according to the manufacturers' instructions. Relative transcript quantification was obtained by qPCR with the transcript-specific primers (Table S2) using PowerUp SYBR Green master mix (Thermo Fisher) on a QuantStudio3 PCR system (Thermo Fisher) or a qTOWER Iris qPCR device (Analytik Jena). Ct values were obtained using the QuantStudio or qTOWER Iris software and averaged across technical replicates. The transcript levels were normalized to the levels of a housekeeping gene *GAPDH* (human) or *R*plp*0* (mouse). The oligonucleotides used for the analysis in human THP-1, DANG, PANC1 and HPAC cells were: *IFNB1* forward ACGCCGCATTGACCATCTAT, *IFNB1* reverse GTCTCATTCCAGCCAGTGCTA, *CXCL10* forward AAGTGGCATTCAAGGAGTACCT, *CXCL10* reverse GGACAAAATTGGCTTGCAGGA, *GAPDH* forward GATTCCACCCATGGCAAATTC, *GAPDH* reverse AGCATCGCCCCACTTGATT. The oligonucleotides used for the analysis in mouse pancreatic cancer 53074 cells were: *Rplp0* forward GGATCTGCTGCATCTGCTTG, *Rplp0* reverse GCGACCTGGAAGTCCAACTA, *Ifnb1* forward CGGAGAAGATGCAGAAGAGT, *Ifnb1* reverse TCAAGTGGAGAGCAGTTGAG.

#### RNA-seq library preparation, sequencing and data processing

Library preparation for bulk-sequencing of poly(A)-RNA was done as described previously.^138^ Barcoded cDNA of each sample was generated with a Maxima RT polymerase (Thermo Fisher) using oligo-dT primer containing barcodes, unique molecular identifiers (UMIs) and an adaptor. 5′ Ends of the cDNAs were extended by a template switch oligo (TSO) and full-length cDNA was amplified with primers binding to the TSO-site and the adaptor. NEB Ultra II FS kit was used to fragment cDNA. After end repair and A-tailing a TruSeq adapter was ligated and 3′-end-fragments were finally amplified using primers with Illumina P5 and P7 overhangs. The library was sequenced on a NextSeq1000 (Illumina) with 65 cycles for the cDNA in read1 and 19 cycles for the barcodes and UMIs in read2. Data was processed using the published Drop-seq pipeline (v1.12) to generate sample- and gene-wise UMI tables.^129^ Reference genome (GRCh38) was used for alignment. Transcript and gene definitions were used according to GENCODE v38.

Raw counts were used for differential expression analysis with DESeq2 (v1.42.1) package.^131^ For plotting of PCA plots and heatmaps counts were transformed using variance-stabilized transformation. Differentially expressed genes were defined as log2 fold change ≤ −1 or ≥ +1 and adjusted *p* value ≤0.05. Pathway analysis was performed using DAVID analysis tool.^139^

#### Transcription factor (TF) motif analysis

TF motif enrichment for known motifs was performed using HOMER package.^133^ Command *findMotifs.pl* was used and a list of gene symbols was supplied as an input. Motifs were searched in the region 400 bp upstream and 100 bp downstream of the TSS by specifying parameters *-start -400 -end 100*. For presentation of enriched TF motifs results from known motifs were used.

### Quantification and statistical analysis

Statistical significance was calculated as indicated in Figure Legends and represented as ns *p* > 0.05, ∗ *p* ≤ 0.05, ∗∗ *p* ≤ 0.01, ∗∗∗ *p* ≤ 0.001, and ∗∗∗∗ *p* ≤ 0.0001 using GraphPad Prism 10.3.1. and RStudio 2024.09.1 + 394.
