# Supplementary material for: STING agonist profiling by nucleotide library defines structural determinants of cyclic dinucleotide recognition
Source: iScience. 2026 Aug 13;29(9):117179. doi: 10.1016/j.isci.2026.117179 (PMC13499098; doi:10.1016/j.isci.2026.117179)
Supplement: Supplementary file 1 [file mmc1.pdf]

iScience, Volume *29*

Supplemental information

STING agonist profiling by nucleotide library defines structural determinants of cyclic dinucleotide recognition

Indra Bekere, Yuliia Hubarzhevska, Sabrina V. Egender, Patrick K. Quoika, Rupert Öllinger, Marie Rose Schrimpf, Roland Rad, Martin Zacharias, and Carina C. de Oliveira Mann

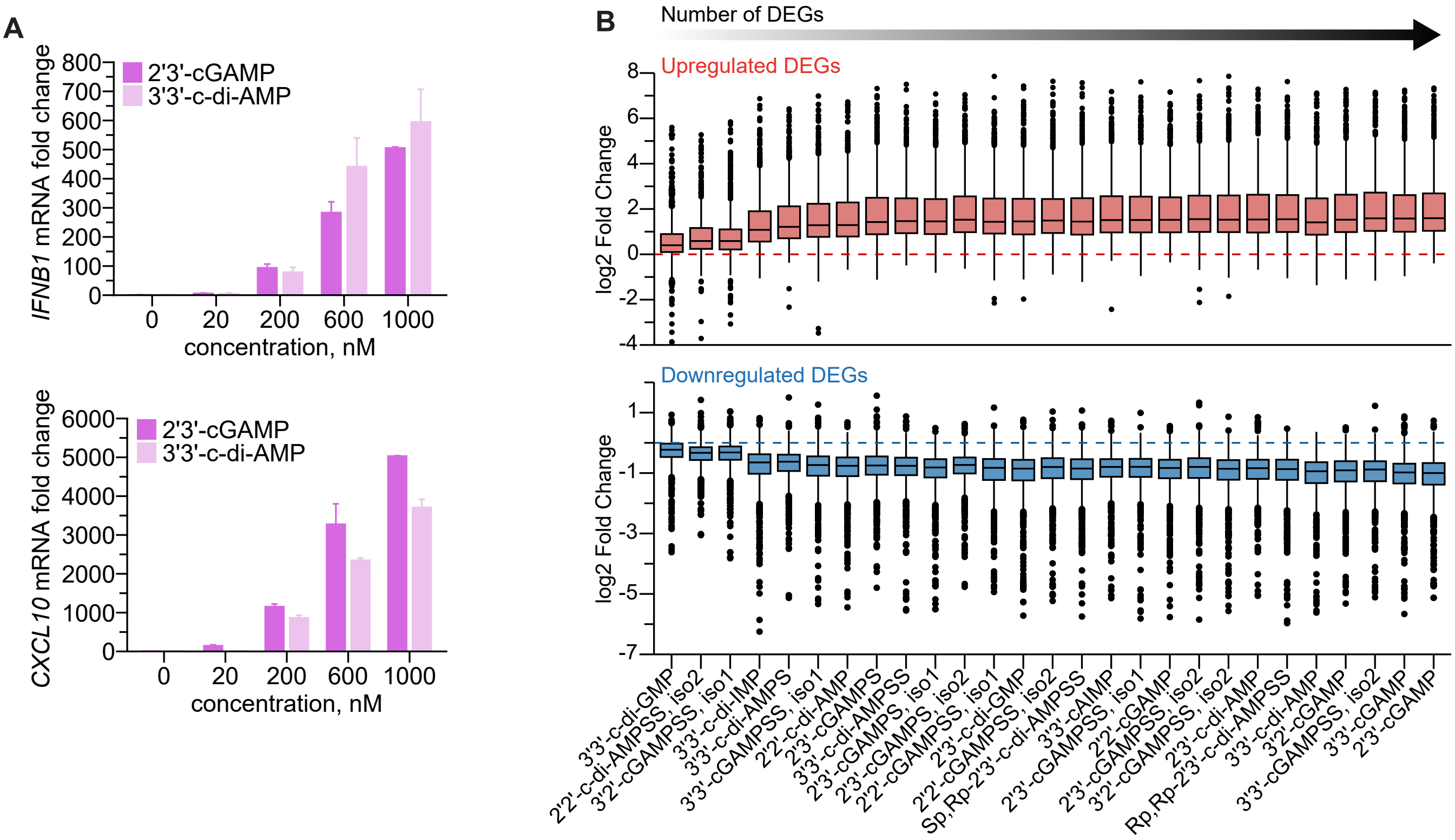

Figure S1. Analysis of nucleotide signaling in THP-1 cells, related to Figure 1.
(A) RT-qPCR analysis of *IFNB1* and *CXCL10* expression after electroporation of THP-1 monocytes with indicated amount of 2′3′-cGAMP and 3′3′-c-di-AMP for 6 h. Bars represent means from two replicates and error bars represent standard deviation.
(B) Boxplots showing log2 fold change for each CDN that induced signaling for upregulated and downregulated DEGs when pooling all DEGs from 27 signaling CDNs together. CDNs are sorted by the number of DEGs that they induced. Boxes encompass the twenty-fifth to seventy-fifth percentile changes. Whiskers extend to the tenth and ninetieth percentiles. Outliers are depicted with black dots. The central horizontal bar indicates the median.

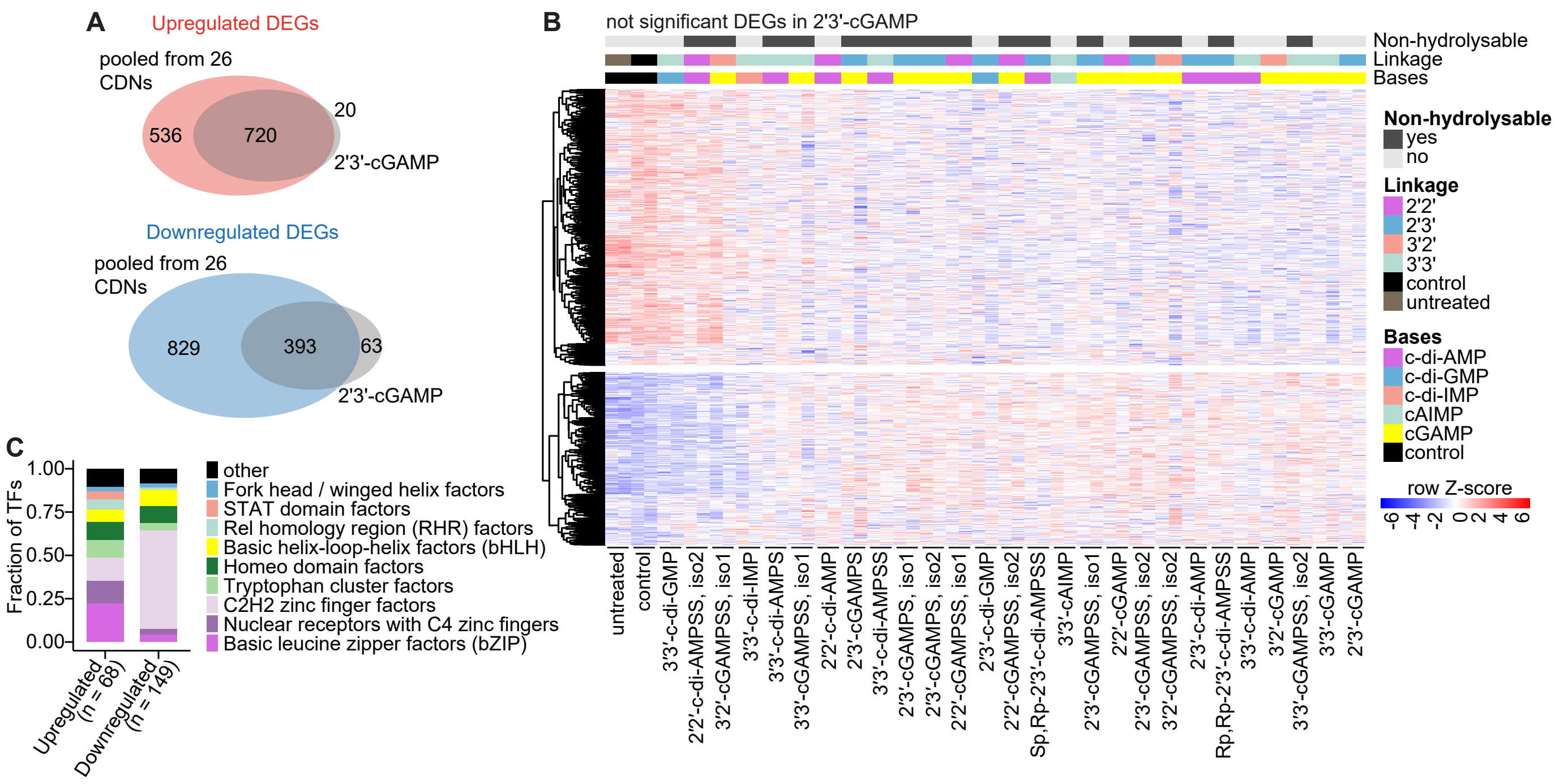

Figure S2. Analysis of gene expression changes that are induced by CDNs, related to Figure 2.

(A) Overlap of upregulated and downregulated DEGs between 2′3′-cGAMP and all the DEGs pooled from the 26 other CDNs, which induced signaling.

(B) Heatmap showing clustering of all DEGs from (A), which were not DEGs and were not significantly regulated by 2′3′-cGAMP (536 upregulated and 829 downregulated genes). Treatments are sorted by the number of DEGs, which they induced. Normalized transcript counts from variance stabilizing transformation (vst) were used, counts are row scaled.

(C) Bar plot showing transcription factor families in the upregulated and downregulated genes from Fig 2C belonging to regulation of transcription based on the analysis with TFcheckpoint database.

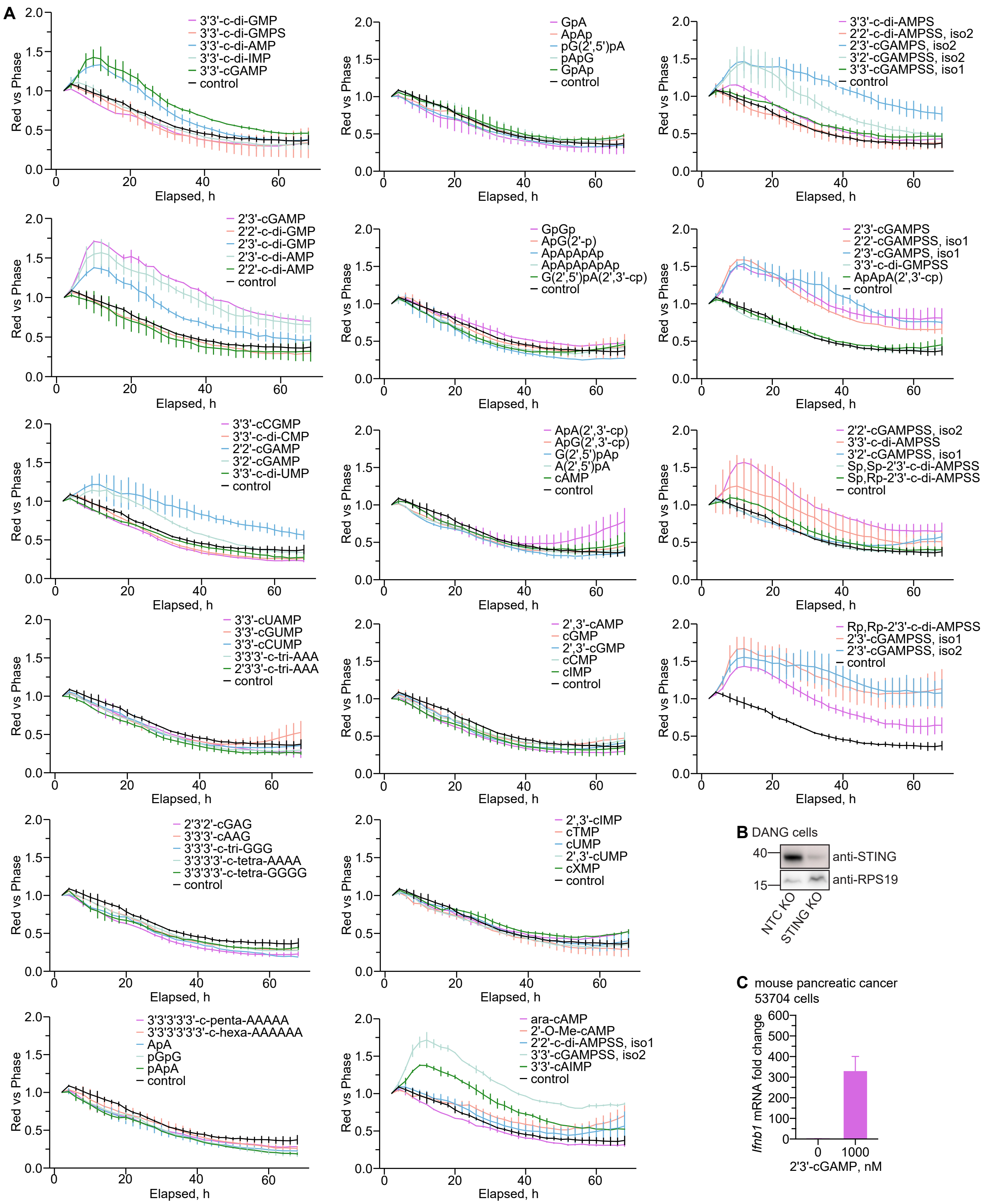

Figure S3. Analysis of nucleotide signaling in pancreatic cancer cells, related to Figure 3.
(A) Line plots showing induction of cell death for each nucleotide after electroporation of nucleotide library in human pancreatic cancer DANG cells. Cell death was quantified as red signal from YOYO-3 fluorescence vs phase. Lines represent means from two replicates, error bars indicate standard deviation. For all conditions red vs phase signal reduces over time due to cell attachment and recovery after electroporation.
(B) Western Blot analysis showing STING levels in DANG NTC KO or STING KO cells with anti-RPS19 as a loading control.
(C) RT-qPCR analysis of *Ifnb1* expression after electroporation of mouse pancreatic cancer cells with 1000 nM of 2′3′-cGAMP for 6 h. Bars represent means from two independent replicates and error bars represent standard deviation.

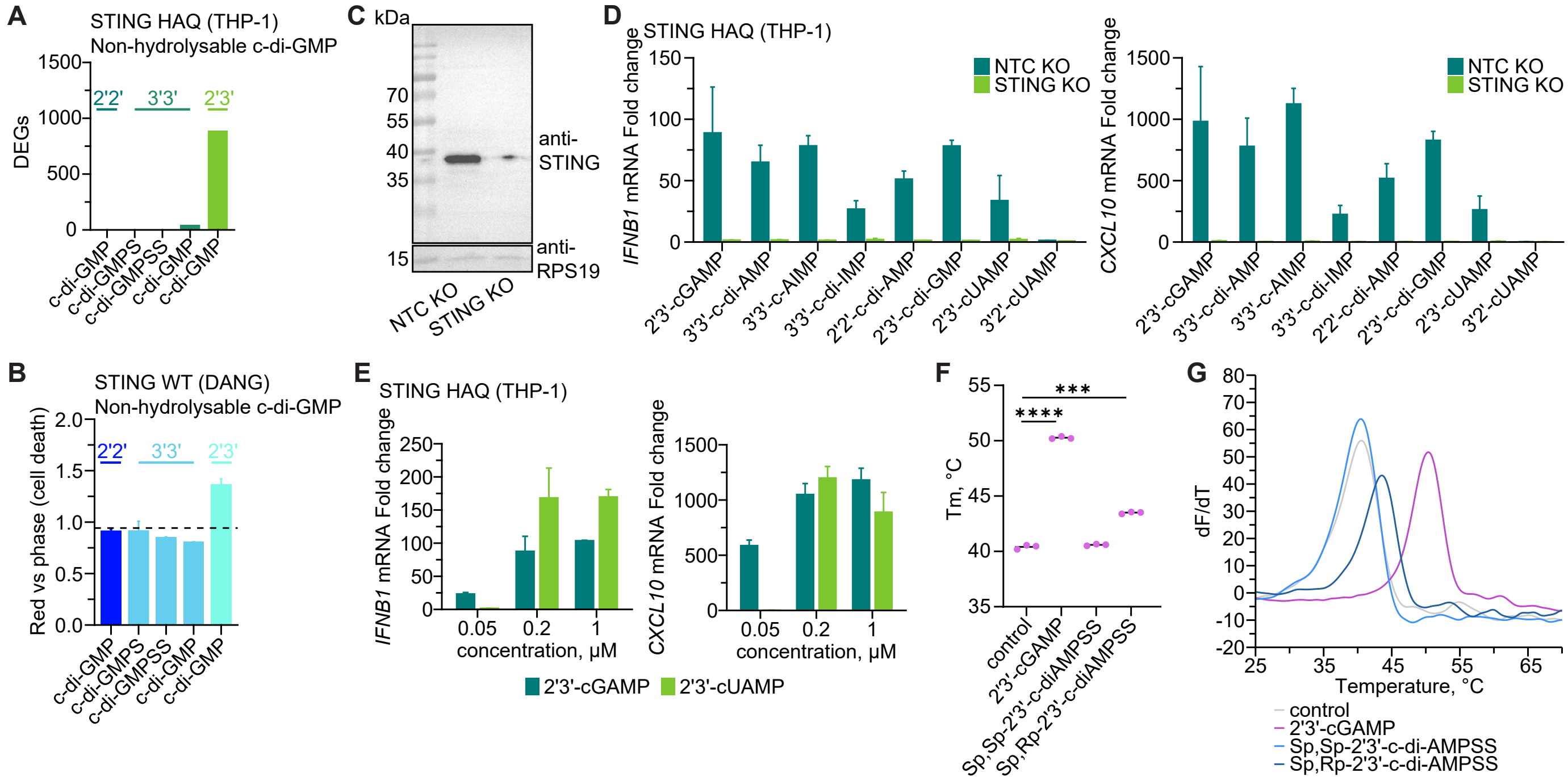

Figure S4. Analysis of STING nucleotide preferences, related to Figure 4.

(A) and (B) Bar plots showing number of DEGs in THP-1 cells (A) and red vs phase (cell death) in DANG cells (B) for all hydrolysable and non-hydrolysable c-di-GMP analogues in the library. In (B) dashed line represents levels of red vs phase (cell death) after control electroporation with water.

(C) Western Blot analysis showing STING levels in THP-1 non-targeting control (NTC) KO or STING KO cells.

(D) RT-qPCR analysis of *IFNB1* and *CXCL10* expression after electroporation of THP-1 NTC KO or STING KO cells with 600 nM of indicated nucleotides for 6 h.

(E) RT-qPCR analysis of *IFNB1* and *CXCL10* expression after electroporation of THP-1 monocytes with 50 nM, 200 nM or 1000 nM of 2′3′-cGAMP or 2′3′-cUAMP for 6 h.

(F) Melting temperature analysis of recombinantly purified STING ligand binding domain with water (control), 2′3′-cGAMP, Sp,Sp-2′3′-c-di-AMPSS and and Sp,Rp-2′3′-c-di-AMPSS by thermal shift assays. Dots: independent replicates, horizontal lines: mean. Paired t-test *** $p \leq 0.001$, **** $p \leq 0.0001$.

(G) Derivative curves from thermal shift assays of a representative experiment from (F).

In (B), (D) and (E) bars represent means and error bars represent standard deviation from at least two experiments.

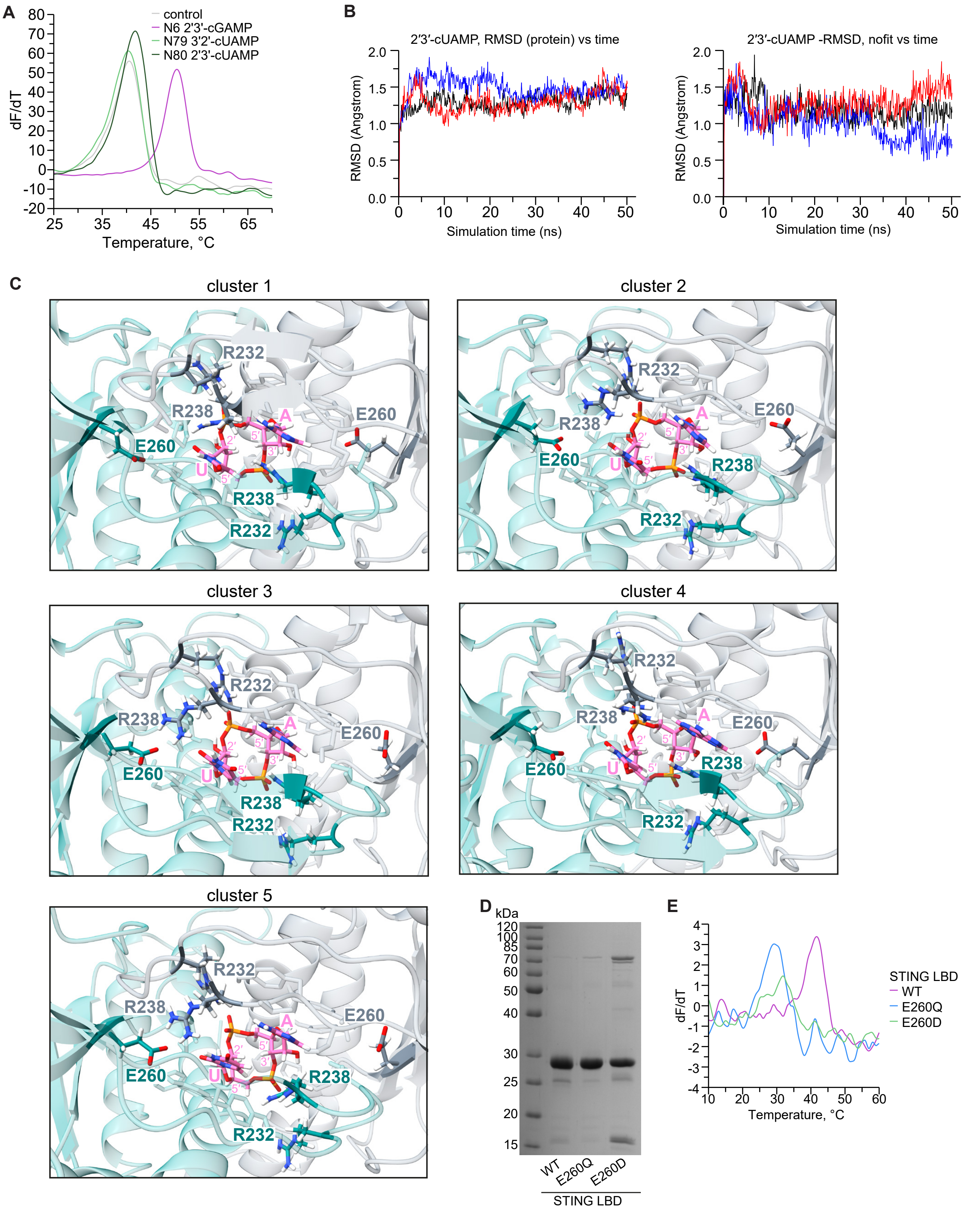

Figure S5. Analysis of STING interaction with 2′3′-cUAMP, related to Figure 5.
(A) Derivative curves from thermal shift assays of a representative experiment from Fig 5A.
(B) (left panel) Root mean square deviation (RMSD) of the protein backbone from the starting reference structure vs. simulation time of the STING dimer in complex with 2′3′-cUAMP. The three different line colors (black, red, blue) correspond to results of 3 independent simulations. (right panel) RMSD of the 2′3′-cUAMP ligand (all non-hydrogen atoms) vs. simulation time after the best superposition of the protein backbone on the reference structure indicating small deviations of the ligand relative to the protein structure.

(C) Structural snap shots representing conformational clusters observed during the MD simulations of STING (cartoon) in complex with 2′3′-cUAMP (stick model). Important 2′3′-cUAMP coordinating residues are labelled and indicated as stick models. The snapshots of clusters 3 and 5 indicate transient contacts of R238 with E260.
(D) SDS-PAGE analysis of recombinantly purified STING ligand-binding domain (LBD) WT, E260Q and E260Q.
(E) Derivative curves from thermal shift assays representing protein melting temperature of recombinantly purified STING ligand-binding domain (LBD) WT, E260Q and E260Q.

Table S1. List of nucleotides in the library and number of DEGs induced by each nucleotide in RNA-seq in THP-1 cells.

Catalog #	Compund Full Name	Number	Short Name	Type	Datasheet	Number of DEGs RNA-seq
C 057-01	Cyclic diguanosine monophosphate (c-diGMP), sodium salt	1	3'3'-c-di-GMP	cyclic dinucleotide	C 057	43
C 125-01	Cyclic diguanosine monophosphorothioate, Sp- isomer (Sp-c-diGMPS), sodium salt	2	3'3'-c-di-GMPS	cyclic dinucleotide	C 125	0
C 088-01	Cyclic diadenosine monophosphate (c-diAMP), sodium salt	3	3'3'-c-di-AMP	cyclic dinucleotide	C 088	1018
C 105-01	Cyclic diinosine monophosphate (c-diIMP), sodium salt	4	3'3'-c-di-IMP	cyclic dinucleotide	C 105	500
C 117-005	Cyclic (adenosine monophosphate- guanosine monophosphate) (c-(ApGp) / 3'3'-cGAMP / c[A(3',5')pG(3',5')p] / c[G(3',5')pA(3',5')p]), sodium salt	5	3'3'-cGAMP	cyclic dinucleotide	C 117	1133
C 161-005	Cyclic (guanosine- (2' −> 5')- monophosphate- adenosine- (3' −> 5')- monophosphate) (c[G(2',5')pA(3',5')p] / cGAMP(2'-5') / 2'3'-cGAMP / 2',5'-3',5'-cGAMP), sodium salt	6	2'3'-cGAMP	cyclic dinucleotide	C 161	1196
C 162-01	Cyclic diguanosine- (2' −> 5')- monophosphate / cyclic bis (2' −> 5') diguanylic acid (c[G(2',5')pG(2',5')p] / 2',5'-2',5'-c-diGMP / c-diGMP(2'-5')), sodium salt	7	2'2'-c-di-GMP	cyclic dinucleotide	C 162	0
C 182-005	Cyclic (guanosine- (2' −> 5')- monophosphate- guanosine- (3' −> 5')- monophosphate) (c[G(2',5')pG(3',5')p] / 2'3'-c-diGMP / 2',5'-3',5'-c-diGMP), sodium salt	8	2'3'-c-di-GMP	cyclic dinucleotide	C 182	887
C 187-005	Cyclic (adenosine- (2' −> 5')- monophosphate- adenosine- (3' −> 5')- monophosphate) (c[A(2',5')pA(3',5')p] / 2'3'-c-diAMP / 2',5'-3',5'-c-diAMP), sodium salt	9	2'3'-c-di-AMP	cyclic dinucleotide	C 187	978
C 188-005	Cyclic (adenosine- (2' −> 5')- monophosphate- adenosine- (2' −> 5')- monophosphate) (c[A(2',5')pA(2',5')p] / 2'2'-c-diAMP / 2',5'-2',5'-c-diAMP), sodium salt	10	2'2'-c-di-AMP	cyclic dinucleotide	C 188	742
C 199-005	Cyclic (cytidine monophosphate- guanosine monophosphate) (c-(CpGp) / c[C(3',5')pG(3',5')p]), sodium salt	11	3'3'-cCGMP	cyclic dinucleotide	C 199	0
C 200-01	Cyclic dicytidine monophosphate (c-diCMP)	12	3'3'-c-di-CMP	cyclic dinucleotide	C 200	0
C 210-005	Cyclic (adenosine- (2' −> 5')- monophosphate- guanosine- (2' −> 5')- monophosphate) (c[A(2',5')pG(2',5')p] / 2'2'-cGAMP / 2',5'-2',5'-cGAMP), sodium salt	13	2'2'-cGAMP	cyclic dinucleotide	C 210	946
C 238-005	Cyclic (adenosine- (2' −> 5')- monophosphate- guanosine- (3' −> 5')- monophosphate) (c[A(2',5')pG(3',5')p] / 3'2'-cGAMP / 3',5'-2',5'-cGAMP), sodium salt	14	3'2'-cGAMP	cyclic dinucleotide	C 238	1024
C 256-01	Cyclic diuridine monophosphate (c-diUMP)	15	3'3'-c-di-UMP	cyclic dinucleotide	C 256	0
C 357-005	Cyclic (adenosine monophosphate- uridine monophosphate) (c-(ApUp) / c[A(3',5')pU(3',5')p] / cUMP-AMP / cUA), sodium salt	16	3'3'-cUAMP	cyclic dinucleotide	C 357	0
C 371-005	Cyclic (guanosine monophosphate- uridine monophosphate) (c-(GpUp) / c[G(3',5')pU(3',5')p] / cGMP-UMP), sodium salt	17	3'3'-cGUMP	cyclic dinucleotide	C 371	0
C 375-005	Cyclic (cytidine monophosphate- uridine monophosphate) (c-(CpUp) / c[C(3',5')pU(3',5')p] / cCMP-UMP), sodium salt	18	3'3'-cCUMP	cyclic dinucleotide	C 375	0
C 362-005	Cyclic triadenosine monophosphate (c-triAMP / c-tri-AMP / cyclic triadenylate / c-A3 / cA3 / cOA3), sodium salt	19	3'3'3'-c-tri-AAA	cyclic oligonucleotide	C 362	0
C 372-005	Cyclic (adenosine- (2' −> 5')- monophosphate- adenosine- (3' −> 5')- monophosphate- adenosine- (3' −> 5')- monophosphate) (c[A(2',5')pA(3',5')pA(3',5')p])	20	2'3'3'-c-tri-AAA	cyclic oligonucleotide		0
C 336-005	Cyclic (guanosine- (2' −> 5')- monophosphate- adenosine- (3' −> 5')- monophosphate- guanosine- (2' −> 5')- monophosphate) (c[G(2',5')pA(3',5')pG(2',5')p])	21	2'3'2'-cGAG	cyclic oligonucleotide		0
C 361-005	Cyclic (adenosine- (3' −> 5')- monophosphate- adenosine- (3' −> 5')- monophosphate- guanosine- (3' −> 5')- monophosphate) (c[A(3',5')pA(3',5')pG(3',5')p] / Cyclic AMP-AMP-GMP / cAAG / 3'3'3'-cAAG), sodium salt	22	3'3'3'-cAAG	cyclic oligonucleotide	C 361	0
C 367-005	Cyclic triguanosine monophosphate (c-triGMP / c-tri-GMP / c[G(3',5')pG(3',5')pG(3',5')p] / cyclic triguanylate / c-G3), sodium salt	23	3'3'3'-c-tri-GGG	cyclic oligonucleotide	C 367	0
C 335-005	Cyclic tetraadenosine monophosphate (c-tetraAMP / c-tetra-AMP / cyclic tetraadenylate / c-A4 / cA4 / cOA4), sodium salt	24	3'3'3'3'-c-tetra-AAAA	cyclic oligonucleotide	C 335	0
C 359-005	Cyclic tetraguanosine monophosphate (c-tetraGMP / c-tetra-GMP / cyclic tetraguanylate / c-G4 / cG4), sodium salt	25	3'3'3'3'-c-tetra-GGGG	cyclic oligonucleotide	C 359	2
C 394-005	Cyclic pentaadenosine monophosphate (c-pentaAMP / c-penta-AMP / cyclic pentaadenylate / c-A5 / cA5 / cOA5), sodium salt	26	3'3'3'3'3'-c-penta-AAAAA	cyclic oligonucleotide	C 394	0
C 332-005	Cyclic hexaadenosine monophosphate (c-hexaAMP / c-hexa-AMP / cyclic hexaadenylate / c-A6 / cA6 / cOA6), sodium salt	27	3'3'3'3'3'3'-c-hexa-AAAAAA	cyclic oligonucleotide	C 332	0
A 083-05	Adenylyl- (3' −> 5')- adenosine (ApA)	28	ApA	linear nucleotide	A 083	1
P 023-01	5'- Phosphoguanylyl- (3' −> 5')- guanosine (pGpG / 5'-p(rG)p(rG)), sodium salt	29	pGpG	linear nucleotide	P 023	0
P 033-01	5'- Phosphoadenylyl- (3' −> 5')- adenosine (pApA / 5'-p(rA)p(rA)), sodium salt	30	pApA	linear nucleotide	P 033	0
G 040-01	Guanylyl- (3' −> 5')- adenosine (GpA)	31	GpA	linear nucleotide		0
A 261-01	Adenylyl- (3' −> 5')- adenosine- 3'- O- phosphate (ApAp)	32	ApAp	linear nucleotide		0
P 081-01	5'- Phosphoguanylyl- (2' −> 5')- adenosine (pG(2',5')pA / 5'-p(rG)(2',5')p(rA)), sodium salt	33	pG(2',5')pA	linear nucleotide	P 081	0
P 082-01	5'- Phosphoadenylyl- (3' −> 5')- guanosine (pApG / 5'-p(rA)p(rG)), sodium salt	34	pApG	linear nucleotide	P 082	0
G 056-01	Guanylyl- (3' −> 5')- adenosine- 3'- O- phosphate (GpAp / 5'-(rG)p(rA)p-3' / 3'5'-GpAp), sodium salt	35	GpAp	linear nucleotide	G 056	0
G 057-01	Guanylyl- (3' −> 5')- guanosine- 3'- O- phosphate (GpGp)	36	GpGp	linear nucleotide		0
A 398-01	Adenylyl- (3' −> 5')- guanosine- 2'- O- phosphate (ApG(2'-p))	37	ApG(2'-p)	linear nucleotide		0
A 423-005	Adenylyl- (3' −> 5')- adenylyl- (3' −> 5')- adenylyl- (3' −> 5')- adenosine- 3'- O- phosphate (ApApApAp)	38	ApApApAp	linear nucleotide		0
A 424-005	Adenylyl- (3' −> 5')- adenylyl- (3' −> 5')- adenylyl- (3' −> 5')- adenylyl- (3' −> 5')- adenosine- 3'- O- phosphate (ApApApApAp)	39	ApApApApAp	linear nucleotide		0
G 061-005	Guanylyl- (2' −> 5')- adenosine- 2', 3'- cyclic monophosphate (G(2',5')pA(2',3'-cp))	40	G(2',5')pA(2',3'-cp)	linear nucleotide		0
A 399-005	Adenylyl- (3' −> 5')- adenosine- 2', 3'- cyclic monophosphate (ApA(2',3'-cp))	41	ApA(2',3'-cp)	linear nucleotide		0
A 407-005	Adenylyl- (3' −> 5')- guanosine- 2', 3'- cyclic monophosphate (ApG(2',3'-cp))	42	ApG(2',3'-cp)	linear nucleotide		0
G 062-01	Guanylyl- (2' −> 5')- adenosine- 3'- O- phosphate (G(2',5')pAp)	43	G(2',5')pAp	linear nucleotide		0
A 352-01	Adenylyl- (2' −> 5')- adenosine (A(2',5')pA)	44	A(2',5')pA	linear nucleotide		0
A 001-500 S	Adenosine- 3', 5'- cyclic monophosphate (cAMP)	45	cAMP	cyclic mononucleotides	A 001	0
A 307-50	Adenosine- 2', 3'- cyclic monophosphate (2',3'-cAMP)	46	2',3'-cAMP	cyclic mononucleotides	A 307	0
G 001-100	Guanosine- 3', 5'- cyclic monophosphate (cGMP)	47	cGMP	cyclic mononucleotides	G 001	0
G 025-50	Guanosine- 2', 3'- cyclic monophosphate (2',3'-cGMP)	48	2',3'-cGMP	cyclic mononucleotides	G 025	0
C 001-50	Cytidine- 3', 5'- cyclic monophosphate (cCMP)	49	cCMP	cyclic mononucleotides	C 001	0
I 001-50	Inosine- 3', 5'- cyclic monophosphate (cIMP)	50	cIMP	cyclic mononucleotides	I 001	0
I 032-05	Inosine- 2', 3'- cyclic monophosphate (2',3'-cIMP)	51	2',3'-cIMP	cyclic mononucleotides	I 032	0
T 005-05	Thymidine- 3', 5'- cyclic monophosphate (cTMP)	52	cTMP	cyclic mononucleotides	T 005	0
U 001-50	Uridine- 3', 5'- cyclic monophosphate (cUMP)	53	cUMP	cyclic mononucleotides	U 001	0
U 004-50	Uridine- 2', 3'- cyclic monophosphate (2',3'-cUMP)	54	2',3'-cUMP	cyclic mononucleotides	U 004	0
X 001-05	Xanthosine- 3', 5'- cyclic monophosphate (cXMP)	55	cXMP	cyclic mononucleotides	X 001	0
A 076-05	Arabinoadenosine- 3', 5'- cyclic monophosphate (ara-cAMP)	56	ara-cAMP	cyclic mononucleotides	A 076	0
M 050-05	2'- O- Methyladenosine- 3', 5'- cyclic monophosphate (2'-O-Me-cAMP)	57	2'-O-Me-cAMP	cyclic mononucleotides	M 050	0
C 229-001	Cyclic diadenosine- (2' −> 5')- monophosphorodithioate, isomer 1 (c[A(2',5')pS-A(2',5')pS] / 2'2'-c-diAMPSS / 2',5'- 2',5'-c-diAMPSS / c-diAMPS)	58	2'2'-c-di-AMPSS, iso1	cyclic dinucleotide		0
C 216-001	Cyclic (adenosine monophosphorothioate- guanosine monophosphorothioate), isomer 2 (c-(ApS-GpS) / 3'3'- cGAMPSS / c[A(3',5')pS-G(3',5')pS]), sodium salt	59	3'3'-cGAMPSS, iso2	cyclic dinucleotide	C 216	1050
C 123-001	Cyclic (adenosine monophosphate- inosine monophosphate) (c-(ApIp) / 3',3'-cAIMP / c-AMP-IMP), sodium salt	60	3'3'-cAIMP	cyclic dinucleotide	C 123	906
C 122-001	Cyclic diadenosine monophosphorothioate, Sp- isomer (Sp-c- diAMPS), sodium salt	61	3'3'-c-di-AMPS	cyclic dinucleotide	C 122	521
C 230-001	Cyclic diadenosine- (2' −> 5')- monophosphorodithioate, isomer 2 (c[A(2',5')pS-A(2',5')pS] / 2'2'-c-diAMPSS / 2',5'- 2',5'-c-diAMPSS / c-diAMPS)	62	2'2'-c-di-AMPSS, iso2	cyclic dinucleotide		84
C 209-001	Cyclic (guanosine- (2' −> 5')- monophosphorothioate- adenosine- (3' −> 5')- monophosphate), isomer 2 (c[G(2',5')pS-A(3',5')p] / cGAMPS(2'-5') / 2'3'-cGAMPS / 2',5'- 3',5'-cGAMPS), sodium salt	63	2'3'-cGAMPS, iso2	cyclic dinucleotide		850
C 247-001	Cyclic (adenosine- (2' −> 5')- monophosphorothioate- guanosine- (3' −> 5')- monophosphorothioate), isomer 2 (c[A(2',5')pS-G(3',5')pS] / 3'2'-cGAMPSS / 3',5'-2',5'- cGAMPSS), sodium salt	64	3'2'-cGAMPSS, iso2	cyclic dinucleotide	C 247	963
C 215-001	Cyclic (adenosine monophosphorothioate- guanosine monophosphorothioate), isomer 1 (c-(ApS-GpS) / 3'3'- cGAMPSS / c[A(3',5')pS-G(3',5')pS]), sodium salt	65	3'3'-cGAMPSS, iso1	cyclic dinucleotide	C 215	638
C 204-001	Cyclic (guanosine- (2' −> 5')- monophosphate- adenosine- (3' −> 5')- monophosphorothioate), (c[G(2',5')pA(3',5')pS] / cGAMPS(2'-5') / 2'3'-cGAMPS / 2',5'- 3',5'-cGAMPS), sodium salt	66	2'3'-cGAMPS	cyclic dinucleotide		745
C 231-001	Cyclic (adenosine- (2' −> 5')- monophosphorothioate- guanosine- (2' −> 5')- monophosphorothioate), isomer 1 (c[A(2',5')pS-G(2',5')pS] / 2'2'-cGAMPSS / 2',5'-2',5'- cGAMPSS), sodium salt	67	2'2'-cGAMPSS, iso1	cyclic dinucleotide		857
C 203-001	Cyclic (guanosine- (2' −> 5')- monophosphorothioate- adenosine- (3' −> 5')- monophosphate), isomer 1 (c[G(2',5')pS-A(3',5')p] / cGAMPS(2'-5') / 2'3'-cGAMPS / 2',5'- 3',5'-cGAMPS), sodium salt	68	2'3'-cGAMPS, iso1	cyclic dinucleotide		840
C 130-001	Cyclic diguanosine monophosphorodithioate, Rp- / Sp- isomers (Rp,Sp-c-diGMPSS), sodium salt	69	3'3'-c-di-GMPSS	cyclic dinucleotide	C 130	0
A 425-001	Adenylyl- (3' −> 5')- adenylyl- (3' −> 5')- adenosine- 2', 3'- cyclic monophosphate (ApApA(2',3'-cp)), sodium salt	70	ApApA(2',3'-cp)	linear nucleotide		0
C 232-001	Cyclic (adenosine- (2' −> 5')- monophosphorothioate- guanosine- (2' −> 5')- monophosphorothioate), isomer 2 (c[A(2',5')pS-G(2',5')pS] / 2'2'-cGAMPSS / 2',5'-2',5'- cGAMPSS), sodium salt	71	2'2'-cGAMPSS, iso2	cyclic dinucleotide		893
C 119-001	Cyclic diadenosine monophosphorodithioate, Rp- / Sp- isomers (Rp,Sp-c-diAMPSS), sodium salt	72	3'3'-c-di-AMPSS	cyclic dinucleotide	C 119	817
C 246-001	Cyclic (adenosine- (2' −> 5')- monophosphorothioate- guanosine- (3' −> 5')- monophosphorothioate), isomer 1 (c[A(2',5')pS-G(3',5')pS] / 3'2'-cGAMPSS / 3',5'-2',5'- cGAMPSS), sodium salt	73	3'2'-cGAMPSS, iso1	cyclic dinucleotide	C 246	101
C 283-001	Cyclic (adenosine- (2' −> 5')- monophosphorothioate[Sp]- adenosine- (3' −> 5')- monophosphorothioate[Sp]) (c[A(2',5')pS[Sp]-A(3',5')pS[Sp]]), sodium salt	74	Sp,Sp-2'3'-c-di-AMPSS	cyclic dinucleotide		0
C 223-001	Cyclic (adenosine- (2' −> 5')- monophosphorothioate[Sp]- adenosine- (3' −> 5')- monophosphorothioate[Rp]) (c[A(2',5')pS[Sp]-A(3',5')pS[Rp]]), sodium salt	75	Sp,Rp-2'3'-c-di-AMPSS	cyclic dinucleotide	C 223	898
C 224-001	Cyclic (adenosine- (2' −> 5')- monophosphorothioate[Rp]- adenosine- (3' −> 5')- monophosphorothioate[Rp]) (c[A(2',5')pS[Rp]-A(3',5')pS[Rp]] / Rp,Rp-2'3'-c-diAMPSS), sodium salt	76	Rp,Rp-2'3'-c-di-AMPSS	cyclic dinucleotide	C 224	995
C201-001 (Set 101)	Cyclic (guanosine- (2' −> 5')- monophosphorothioate- adenosine- (3' −> 5')- monophosphorothioate) (c[G(2',5')pS-A(3',5')pS] / 2'3'-cGAMPSS / 2'3'-cGsAsMP), isomer 1	77	2'3'-cGAMPSS, iso1	cyclic dinucleotide	Set 101	913
C208-001 (Set 101)	Cyclic (guanosine- (2' −> 5')- monophosphorothioate- adenosine- (3' −> 5')- monophosphorothioate) (c[G(2',5')pS-A(3',5')pS] / 2'3'-cGAMPSS / 2'3'-cGsAsMP), isomer 2	78	2'3'-cGAMPSS, iso2	cyclic dinucleotide	Set 101	953
C 398-005	Cyclic (adenosine- (2' −> 5')- monophosphate- uridine- (3' −> 5')- monophosphate) (c[A(2',5')pU(3',5')p] / 2'3'-cAUMP / 2',5'-3',5'-cAUMP), sodium salt		2'3'-cAUMP	cyclic dinucleotide	C 398	NA
C 399-005	Cyclic (uridine- (2' −> 5')- monophosphate- adenosine- (3' −> 5')- monophosphate) (c[U(2',5')pA(3',5')p] / 2'3'-cUAMP / 2',5'-3',5'-cUAMP), sodium salt		3'2'-cAUMP	cyclic dinucleotide	C 399	NA

Table S2. Primers for RT-qPCR

RT-qPCR human *IFNB1* forward	ACGCCGCATTGACCATCTAT
RT-qPCR human *IFNB1* reverse	GTCTCATTCCAGCCAGTGCTA
RT-qPCR human *CXCL10* forward	AAGTGGCATTCAAGGAGTACCT
RT-qPCR human *CXCL10* reverse	GGACAAAATTGGCTTGCAGGA
RT-qPCR human *GAPDH* forward	GATTCCACCCATGGCAAATTC
RT-qPCR human *GAPDH* reverse	AGCATCGCCCCACTTGATT
RT-qPCR mouse *Ifnb1* forward	CGGAGAAGATGCAGAAGAGT
RT-qPCR mouse *Ifnb1* reverse	TCAAGTGGAGAGCAGTTGAG
RT-qPCR mouse *Rplp0* forward	GGATCTGCTGCATCTGCTTG
RT-qPCR mouse *Rplp0* reverse	GCGACCTGGAAGTCCAACTA
